# Microglia and monocytes in inflammatory CNS disease: integrating phenotype and function

**DOI:** 10.1007/s00401-021-02384-2

**Published:** 2021-12-01

**Authors:** Alanna G. Spiteri, Claire L. Wishart, Roger Pamphlett, Giuseppe Locatelli, Nicholas J. C. King

**Affiliations:** 1 Viral Immunopathology Laboratory, Infection, Immunity and Inflammation Research Theme, School of Medical Sciences, Faculty of Medicine and Health, The University of Sydney, Sydney, NSW 2006 Australia; 2 Sydney Cytometry Facility, The University of Sydney and Centenary Institute, Sydney, NSW 2006 Australia; 3 Ramaciotti Facility for Human Systems Biology, The University of Sydney and Centenary Institute, Sydney, NSW 2006 Australia; 4 Charles Perkins Centre, The University of Sydney, Camperdown, NSW 2050 Australia; 5 Brain and Mind Centre, School of Medical Sciences, Faculty of Medicine and Health, The University of Sydney, Camperdown, NSW 2050 Australia; 6 Department of Neuropathology, Royal Prince Alfred Hospital, Camperdown, NSW 2050 Australia; 7 Theodor Kocher Institute, University of Bern, Bern, 3012 Switzerland; 8 The University of Sydney Institute for Infectious Diseases, The University of Sydney, Sydney, NSW 2006 Australia; 9 The University of Sydney Nano Institute, The University of Sydney, Sydney, NSW 2006 Australia

**Keywords:** Microglia, Monocyte-derived cells, Immune-mediated pathology, Neuroinflammation, Neurodegeneration, Encephalitis

## Abstract

In neurological diseases, the actions of microglia, the resident myeloid cells of the CNS parenchyma, may diverge from, or intersect with, those of recruited monocytes to drive immune-mediated pathology. However, defining the precise roles of each cell type has historically been impeded by the lack of discriminating markers and experimental systems capable of accurately identifying them. Our ability to distinguish microglia from monocytes in neuroinflammation has advanced with single-cell technologies, new markers and drugs that identify and deplete them, respectively. Nevertheless, the focus of individual studies on particular cell types, diseases or experimental approaches has limited our ability to connect phenotype and function more widely and across diverse CNS pathologies. Here, we critically review, tabulate and integrate the disease-specific functions and immune profiles of microglia and monocytes to provide a comprehensive atlas of myeloid responses in viral encephalitis, demyelination, neurodegeneration and ischemic injury. In emphasizing the differential roles of microglia and monocytes in the severe neuroinflammatory disease of viral encephalitis, we connect inflammatory pathways common to equally incapacitating diseases with less severe inflammation. We examine these findings in the context of human studies and highlight the benefits and inherent limitations of animal models that may impede or facilitate clinical translation. This enables us to highlight common and contrasting, non-redundant and often opposing roles of microglia and monocytes in disease that could be targeted therapeutically.

## Introduction

**Fig. 1:**
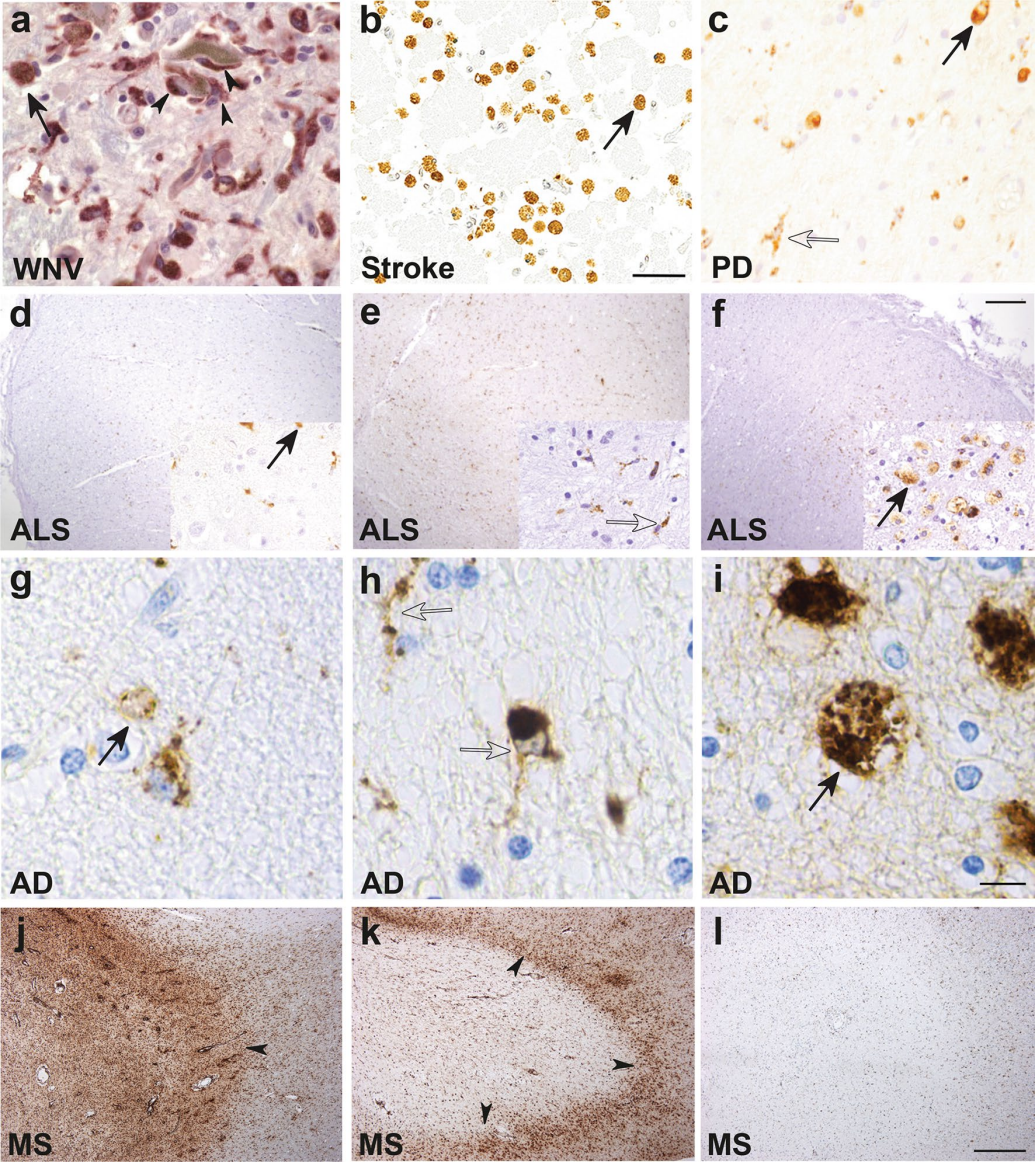
Macrophage/microglial CD68 tissue staining in human CNS pathologies. Ameboid (solid arrows, likely macrophages and/or reactive microglia) and ramified (open arrows, likely microglia) CD68^+^ myeloid cells are shown in various neuropathologies: **a** West Nile virus (WNV) [247]. Macrophage/microglia engulfing a degenerating neuron (arrowheads) in the substantia nigra in a patient with fulminant WNV encephalitis (400X magnification). **b** Cortical stroke [94]. Foamy macrophages/microglia are present in a cerebral infarct (several weeks old) (scale bar represents 50 μm). **c** Parkinson disease (PD) [65]. Ramified microglia and macrophages with enlarged cytoplasm and short stout processes are present in the substantia nigra (400X magnification). **d–f** Amyotrophic lateral sclerosis (ALS) [38]. The three images show the variable extent of microglia activation in the corticospinal tract in patients with ALS assessed as either mild (**d**), moderate (**e**) or severe (**f**) (scale bar represents 250 μm). **g–i** Alzheimer disease (AD) [148]. The three images show the rounded ameboid microglia (**g**), ramified microglia (**h**) or foamy macrophages (**i**) that can be seen in AD brains (scale bar represents 10 μm). **j–l** Multiple sclerosis (MS) [193]. Three images show variable inflammatory activity in MS, with either numerous foamy macrophages within a demyelinating plaque (**j**), macrophages at the rim of a plaque (arrowheads) (**k**), and an inactive plaque with only a few ramified microglia (**l**). All images reproduced with permission

Inflammation of the central nervous system (CNS) is a feature of many neurological disorders including infectious, autoimmune, sterile inflammatory, demyelinating and neurodegenerative diseases [35, 58, 140, 194, 288]. Tissue-resident and infiltrating myeloid cells, such as microglia and monocytes, are recruited to foci of infection, injury or inflammation in many of these CNS pathologies (Fig. 1), suggesting a role for these cells in the pathophysiology of disease [29]. Microglia and monocytes are both members of the mononuclear phagocyte system that carry out essential tissue-specific functions, critical for homeostasis and the response against pathogen evasion [139, 159, 261, 299]. Although the precise contributions of microglia and monocytes to tissue damage and repair in CNS disease remain poorly resolved, they nevertheless represent potential candidates for targeted therapeutics.

Microglia are tissue-resident macrophages of the CNS parenchyma. These cells arise from uncommitted KIT^+^ erythromyeloid precursors [181, 316], which seed the brain from the yolk sac at embryonic day 9.5 in the mouse [126], well before other glial cells and the formation of the blood–brain barrier (BBB) [130, 181]. They are subsequently renewed in situ independently of bone marrow (BM) hematopoietic stem cells (HSC) [3]. However, more recently, other views challenging the sole yolk sac origin of microglia have emerged [55].

In the healthy homeostatic CNS, microglia comprise the predominant myeloid population, followed by non-parenchymal CNS macrophages [182], collectively called CNS- or border-associated macrophages (CAMs or BAMs) [20, 235, 259]. Relative to BAM/CAMs, microglia uniquely express transmembrane protein 119 (TMEM119) [26], hexosaminidase subunit beta (Hexb) [174, 214, 215], P2Y G-protein-coupled 12 (P2RY12) [41], sialic acid-binding immunoglobulin-type lectin H (Siglec-H) and Spalt-like transcription factor 1 (Sall1) [45], express low levels of CD45 compared to leukocytes outside the CNS, and have a distinct morphology and anatomical location [20, 163, 235, 259, 317]. This makes their identification in the homeostatic brain fairly straight-forward. However, during inflammation in response to CNS perturbation, microglia become *reactive* or *activated*, a state in which they upregulate CD45, partially or totally retract their cytoplasmic extensions and increase their somatic volume to adopt a more amoeboid morphology [266, 303]. Microglia are also joined by a substantial infiltrate of BM-derived monocytes [298], both of which similarly express typical myeloid markers [19, 204, 298, 299, 332] (such as CD68, Fig. 1). This hampers the accurate discrimination of these cell types during neuroinflammation in the mouse and human CNS.

Monocytes are peripheral myeloid cells derived from the fetal liver during embryogenesis and are continuously renewed throughout postnatal life from HSCs in the adult BM [115]. In the mouse, ‘inflammatory’ monocytes (Ly6C^hi^) and ‘patrolling’ monocytes (Ly6C^lo^) have been identified [114]. In humans, 'classical' monocytes (equivalent to Ly6C^hi^ monocytes in mice) make up the majority of the circulating monocyte pool, with the remaining portion made up by intermediate and ‘non-classical’ monocytes (Ly6C^lo^ monocytes in mice) [251].

During homeostasis, circulating Ly6C^hi^ monocytes traffic through semi-permeable CNS regions, such as the choroid plexus and dura mater, where they increase the local complexity of BAMs [128, 317]. The BBB prevents the infiltration of monocytes and other peripheral immune cells into the CNS parenchyma under homeostatic conditions [230]. During inflammation, however, BBB breakdown and leakiness permits the infiltration of monocytes into the parenchyma. Such infiltrating monocyte-derived cells (MC), like monocyte-derived macrophages (MDM) and dendritic cells (moDC), can adopt a microglia-like phenotype upon entry into the inflamed brain. While these cells do not necessarily upregulate genes expressed by homeostatic microglia [25, 66], the phenotypic similarities between populations of resident and infiltrating myeloid cells may approximate one another in neuroinflammation, hampering accurate identification of their respective functions in disease [298, 299].

Animal models employing more recent and precise experimental approaches, such as microglia-depletion drugs, cell type-specific markers, gene silencing, lineage tracing, and in vivo imaging techniques, have been used to distinguish microglia and monocytes in various neuropathologies to understand their functional roles [26, 88, 91, 214, 220, 299, 343]. While animal models may fail to fully replicate all aspects of human disease, they have been indispensable in understanding specific pathogenic mechanisms and the effect of therapeutics on particular aspects of disease. We now understand microglia and monocytes to be highly heterogeneous in phenotype and function, having both protective and pathogenic roles that contribute to disease onset, progression and/or recovery.

Here, we integrate and contrast human and animal findings from studies investigating viral encephalitis, CNS injury, neurodegenerative disease and autoimmune neuroinflammation to elucidate key agonistic and antagonistic features of microglia and their BM-derived monocyte counterparts across these neuropathologies. We focus on studies using more selective approaches, including transcriptomic profiling, high-dimensional cytometry and myeloid cell depletion to more accurately dissect their functional roles in neurological disease. In doing so, we identify common pathogenic or protective cell type-specific functions and phenotypes that may aid the development of targeted therapeutics across CNS diseases.

### Viral encephalitis

Neuroinflammation resulting from viral infection of the CNS parenchyma (*i.e.*, viral encephalitis) carries  ~ 5–30% fatality rate, with survivors experiencing severe neurological sequelae and memory deficits that may worsen over time [111, 209, 272, 329, 340]. Upon entry into the parenchyma, viruses infect and replicate in neurons and/or glia, initiating an inflammatory response. The production of various soluble factors by resident brain cells recruits a range of leukocytes from the periphery, including monocytes and lymphocytes, which carry out effector functions necessary for viral clearance [188]. In severe viral encephalitis, large numbers of microglia and peripherally-derived MDMs can be seen in human post-mortem tissue (Fig. 1a). However, this response is not always beneficial and an overexuberant inflammatory response may also drive neuropathology [185]. For instance, in West Nile virus (WNV) encephalitis, the infiltration of MDMs is particularly associated with injury to brain cells, tissue swelling and the development of seizures [120–123]. In modelling these responses in the CNS, murine models inoculated with a relevant neurotropic virus are commonly used, although this may not necessarily reflect true disease in the human.

### Relevance of murine models to human viral encephalitis disease

While extensive research has uncovered various processes involved in the pathogenesis of viral encephalitis, there are significant differences between mouse models and human disease. To begin with, researchers use a variety of inoculation routes for the same virus, including intradermal, subcutaneous, intravenous, intramuscular, intracranial or intranasal. These routes of inoculation model different aspects of pathology, but vary in invasiveness and viral dose, with very few replicating the usual route of infection by the homologous virus in humans [39, 72, 80, 184, 337]. This has a significant impact on (1) local innate and immune defensive responses, (2) spread of virus locally or systemically, and (3) presentation of viral antigen in local draining lymph nodes versus wide dissemination throughout the animal, all of which may substantially alter the outcomes of infection [39, 337].

The choice of mouse and virus strain may further influence disease pathogenesis and the outcome of infection. Investigators use different mouse strains, frequently, C57BL/6 or BALB/c, for example, which have widely disparate responses to viruses. Genetically modified, usually transgenic or knockout animals, are also commonly used, which take little account of the compensatory changes that may occur in the absence or presence of a particular gene and which consequently demonstrate very different immune responses to infection [39, 80], with local availability often dictating the choice of strain for these studies. It is also common for different laboratories to use different strains of the same virus, often a specifically laboratory-adapted strain, often dictated by the availability of the appropriate biosafety facilities (e.g., BSL-2 versus BSL-3), or a virus monogamous to the model host, or one that is seldom encountered by it [141], potentially compounding this still further. Mice are almost always used at an age convenient to the model being studied, with weaning, the formation of the BBB, puberty and sexual maturity, being common temporal landmarks for infection [8, 123, 368]. This is often to enable reliable infection (e.g., some neurotropic viruses will only infect mice prior to the formation of the BBB), but differences in immune system maturity can significantly influence the immune response to infection. Furthermore, until recently in immunological work, it has been common to use only female mice, which ignores the effect of sex differences on the immune response. Finally, there is increasing awareness of the effects of the cellular circadian clock and diet on cellular metabolism driving immune responses [195, 307, 308], with differences in housing conditions and significant variability in macronutrient composition of ad libitum ‘standard’ chow between institutions.

Variables of this kind make infallible comparison between experimental models difficult, even before considering their applicability in humans. However, the stochastic incidence and clinical presentation of established illness and its subsequent chronicity in human disease, often after any antecedent pathogenesis can be recorded, the variable availability and timing of investigative modalities, access to samples, usually restricted to body fluids, occasional biopsies and/or post-mortem tissue, with tissue degradation due to post-mortem delays often reducing the reliability of results, also impose significant limitations on its detailed study, ultimately increasing our reliance on experimental animal models as an important complementary approach to investigating human disease. Needless to say, many of these benefits and drawbacks also apply to the study of other neurological diseases.

### Microglia in viral encephalitis

#### Neuroprotective role of microglia in acute-phase viral encephalitis

Historically, using non-selective techniques, microglia were argued to have a neurotoxic role in encephalitis, producing inflammatory cytokines and orchestrating immune-mediated pathology [53, 263]. With the recent development of PLX5622, a colony-stimulating factor 1 receptor (CSF1R) inhibitor that causes rapid microglia depletion [295], the functions of microglia in viral encephalitis have been extensively revised. Despite other CSF1R inhibitors (i.e., PLX3397 and BLZ945) being available before PLX5622, few studies investigating viral encephalitis utilised these earlier compounds. PLX5622 is generally formulated into chow or administered via oral gavage. It enables sustained microglial depletion without breaching the BBB, without toxicity and without initiating an inflammatory response, as seen with other microglial depletion methods [295, 299, 331]. However, this reagent is not microglia-specific, as other cells also express CSF1R [131]. Despite a lack of confirmatory evidence [134], it was suggested that PLX5622 also causes functional impairments in peripheral lymphoid and myeloid compartments [198]. This molecule likely also affects BAMs, as reported in the case of PLX3397 [317], placing an important caveat on the interpretation of data where PLX5622 is assumed to be purely microglia-specific.

**Fig. 2:**
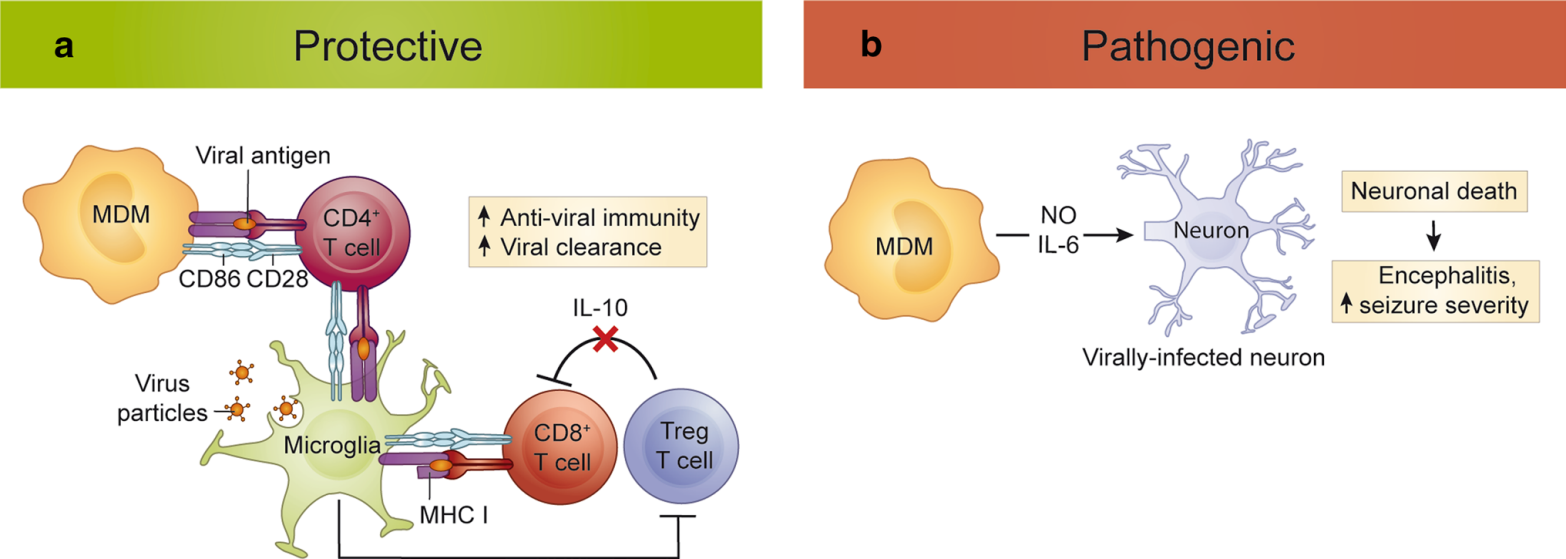
Protective and pathogenic roles of monocytes and microglia in viral encephalitis. **a** Protective functions. In viral encephalitis, microglia enhance viral clearance by phagocytosing virus-infected cells. Both microglia and MDMs stimulate anti-viral T cell responses, which is optimized by microglia-mediated regulation of Treg infiltration. **b** Pathogenic functions. NO- and IL-6-producing MDMs exacerbate neuronal damage and contribute to immunopathology. MDM, monocyte-derived macrophage; MHC, major histocompatibility complex; *NO* nitric oxide; *IL* interleukin; *Treg* regulatory T cell

Microglia ablation in Mouse Hepatitis Virus (MHV), Japanese Encephalitis Virus (JEV), Theiler’s Encephalomyelitis Virus (TMEV), Pseudorabies Virus (PRV) and WNV encephalitis models has demonstrated a clear neuroprotective role of these cells in the acute phase of infection [96, 107, 274, 279, 335, 344], and alternative microglial depletion methods in Vesicular Stomatitis Virus (VSV) infection have produced similar findings [59, 234]. In some of these studies, microglial depletion resulted in increased weight loss, increased viral burden and persistence, as well as increased viral spread in the CNS and into the periphery [96, 107, 335, 344]. A detailed comparison of recent work using PLX5622 to deplete microglia in different viral encephalitis models is shown in Tables 1 and 2. Collectively, these findings suggest that microglia are pivotal in controlling virus spread in the CNS, with this protective role required especially in the earlier phases of disease [96, 107, 335, 344]. Nevertheless, there is substantial disparity between studies in the elucidation of the precise mechanisms by which microglia control viral spread, presumably consistent with the divergent evolution of virus-host survival strategies for different viral pathogens. Figure 2 illustrates the putative pathogenic and protective roles of microglia and MDM in viral encephalitis.

**Table 1:** Comparison of studies using PLX5622 to deplete microglia in various models of viral encephalitis (part 1)

Study	Virus	Infection	# days mice were fed PLX5622 before infection	Mice	Enhanced mortality?	Enhanced morbidity?	Enhanced weight loss?	Enhanced viral load?
**Sanchez et al.** [274]	Daniel's strain of TMEV	intracranial (i.c)	7	C57BL/6 Males 4 weeks old	Yes	Yes	Not recorded (N/R)	N/R
**Funk** **et al.** [107]	WNV-NY, strain 3000.0259	footpad (f.p)	14	C57BL/6 Males 6 weeks old	Yes	Yes	Yes	Yes
		i.c			No	N/R	No	No
	Attenuated WNV-NS5- E218A				Yes	N/R	Yes	Yes
**Wheeler** **et al.** [344]	Neuroattenuated variant of the JHMV strain of MHV	i.c	7	C57BL/6 Males 5–6 weeks old	Yes	N/R	N/R	Yes
	N1347A, an rJ macrodomain point mutant virus	intranasal (i.n)			Yes	N/R	N/R	N/R
	Recombinant parental JHMV	i.p			Yes	N/R	N/R	N/R
**Waltl et al.** [334]	Daniel’s strain of TMEV	i.c	21	JAX® C57BL/6 J (B6) Female 4 weeks old	Yes	Yes	Yes	Yes
**Seitz et al.** [279]	WNV-NY99	f.p	14	Swiss-Webster Female 7–10 weeks old	Yes	N/R	No	Yes
	p3 strain of JEV				Yes	N/R	No	Yes
**Fekete et al.** [96]	PRV-Bartha derivative, PRV-Bartha-Dup-Green	intraperitoneal (i.p.) or directly into the epididymal white adipose tissue	21	C57BL/6 J Gender not specified 12–18 weeks old	N/R	Yes	N/R	Yes

**Table 2:** Comparison of studies using PLX5622 to deplete microglia in various models of viral encephalitis (part 2)

Study	Sanchez et al. [274]	Funk et al. [107]		Wheeler et al. [344]			Waltl et al. [334]	Seitz et al. [279]		Fekete et al. [96]
Virus	Daniel’s strain of TMEV	WNV-NY, strain 3000.0259	Attenuated WNV-NS5- E218A	Neuro-attenuated variant of the JHMV strain of MHV	N1347A, an rJ macrodomain point mutant virus	Recombinant parental JHMV (rJHMV)	Daniel’s strain of TMEV	WNV-NY99	p3 strain of JEV	PRV-Bartha derivative, PRV-Bartha-Dup-Green (BDG)
**Enhanced neuroinflammation/ neurodegeneration?**	Axonal damage and demyelination in spinal cords	N/R	Increased neuronal apoptosis in the hippocampus and cerebellum at dpi 6.	N/R			Increased: neuroinflammation, perivascular infiltrates, astrogliosis and neurodegeneration.	N/R		N/R
**Change in macrophage infiltrate in PLX5622-treated animals?**	N/R	N/R	Decreased numbers of macrophages Reduced CD86 expression.	Increased numbers of macrophages. Reduced expression of MHC-II, increased expression of Ly6C and differential expression of 235 genes.	N/R		No change	N/R		Decreased numbers of macrophages.
**Change in cytokine production in the CNS of PLX562- treated animals?**	N/R	N/R	Decreased RNA: IFN-β, IFN-γ, TNF, NOS2, CD86 & CD68 (brain).	Increased RNA: IFN-β, IL6 at dpi 3 and IFN-α, IFN-β, IL6 at dpi 5. No change in protein: IFN-α, IFN-β & IL6 at dpi 5 (brain).	N/R		Decreased RNA: TGF-β1 (brain). Increased RNA: IL-6 (brain), IL10 (brain) & IFN-γ (brain & spinal cord).	Increased RNA: CCL2 at dpi 6 and CCL2, CCL7, CXCL9 & CXCL10 at dpi 9 (brain).	Increased RNA: CCL3 at dpi 8 (brain).	Decreased protein: IL-1α & RANTES (hypothalamic brain tissue).
**Altered T cell response in the CNS in PLX5622-treated animals?**	N/R	N/R	Increased percentage and number of CD8^+^ T cells. CD8^+^CD45^+^NS4B^+^ T cells showed a decreased frequency of CD69^+^ and CD160^+^ cells and reduced expression of CD69 and CD160. All relative to infected, non-microglia-depleted animals.	Decreased percentage & numbers of CD4^+^ T cells & virus-specific CD4^+^ T cells. Decreased percentage & numbers T regs. All relative to infected, non-microglia-depleted animals.	N/R		Increased T regs in the spinal cord and hippocampus (relative to microglia-depleted, non-infected animals). Decreased numbers CD4^+^ and CD4^+^CD44^+^ T cells in the whole brain (relative to non-depleted, infected animals).	N/R		No change in the number of CD8^+^CD3^+^ T cells in the brain (relative to non-depleted and/or infected animals).
**Systemic responses in PLX5622-treated animals**	N/R	Loss of CD80 (percentage & numbers) & CD86 (percentage & mean fluorescence intensity) expression on MHC-II^+^CD11c^+^CD45^+^ (DCs) cells, increase in the numbers of CD4^+^ and CD8^+^ T cells, no change in CD69^+^ on CD8^+^ CD45^+^ T cells and no change in WNV-specific NS4B^+^ tetramer staining of CD8^+^CD45^+^ cells in the spleen of WNV-NS5- E218A infected, PLX5622-treated animals Decreased CD80 expression on DCs in the blood and decreased CD86 on DCs pDLN at dpi 4 (WNV-NY, f.p.) Reduced numbers of CD45^+^MHC-II^+^CD11c^+^ and CD11b-CD11c^+^ in the blood in non-infected, PLX-treated animals No change in antigen-presenting cell (APC) populations in the spleen or BM in non-infected, PLX5622-treated animals No changes in T cells, CD11b^+^, Ly6C^+^ or Ly6G^+^ cells in spleen, BM or blood in non-infected, PLX5622-treated animals		No change in the number of CD11b^+^ cells in the non-infected spleen No change in the total number of cells on dpi 3 or 5 in the draining cervical lymph nodes (dCLN) in i.c. MHV-infection PLX5622 treatment did not change the number of virus-specific CD4^+^ or CD8^+^ T cells after i.p. infection with rJ			Reduced number of CSF1R monocytes in the blood in PLX5622-treated mock and infected groups compared to non-PLX-treated animals No change CD4^+^ and CD8^+^ ratio in the spleen of PLX5622-treated animals No change in the number Iba1^+^ macrophages in the spleen (by immunohistochemistry)	N/R		PLX5622 did not cause a significant reduction in circulating or splenic myeloid populations: monocytes, granulocytes, macrophages and B cells Increase in circulating granulocytes (non-significant – infected depleted vs infected non-depleted, significant – infected depleted vs non-infected depleted) Decrease in circulating CD8^+^ T cells (non-significant – infected depleted vs infected non-depleted, significant – infected depleted vs non-infected depleted)
**Protective role of microglia**	Microglia mediate antiviral responses in the CNS	Microglia are important in the restimulation of a CD8^+^ T cell in the CNS for an effective antiviral T cell response to control viral spread Lack of virologic control was specifically due to the reduction in APCs (as measured by macrophage and microglial expression of B7 molecules) in the CNS, important for a CD8^+^ T cell response		Microglia are important in the earliest stages of disease, preventing the neurological spread of the virus by restimulating CD4^+^ T cells that enter the brain The absence of microglia was associated with the infiltration of immature macrophages and the reduction in MHC-II in the CNS (on microglia and macrophages), preventing the local reactivation of CD4^+^ T cells for an effective antiviral T cell response			Microglia depletion resulted in increased numbers of T regs and IL-10, suppressing cytotoxic CD8^+^ T cell activity and preventing an effective antiviral T cell response to control viral spread	Microglia mediate antiviral responses in the CNS		The protective role of microglia relies on the rapid and precise migration of microglia to virally infected neurons and the subsequent phagocytosis to prevent viral spread and the presence of viral antigens in the brain

#### Microglial role in effective T cell responses mediating viral clearance

The importance of an effective T cell response in viral clearance and improved disease outcome has been shown in various viral encephalitis models. T cell activation requires antigen presentation and co-stimulation via antigen-presenting cell (APC)-expressed Major Histocompatibility Complex antigen (MHC) and CD80 (B7-1)/CD86 (B7-2), respectively. Strikingly, in the absence of microglia, several studies have shown an ineffective or inadequate CD8^+^, CD4^+^ T cell and/or regulatory T cell (Treg) response, suggesting a role for microglia in T cell infiltration and/or activation. However, many of these studies lack specific evidence and do not take into account the indirect effects caused by microglia-depletion agents.

##### CD8^+^ T cells

A reduction in the number of APCs in the CNS following PLX5622 treatment was thought to contribute to a sub-optimal CD8^+^ T cell response during WNV infection, resulting in poor virus control [107], implicating microglia in CD8^+^ T cell activation (Table 2). However, in this model, both microglia and MDM numbers were reduced in the brain, making it difficult to determine whether microglia or MDMs were responsible for the reduced T cell activation. Paradoxically, numbers of CD8^+^ T cells were increased in the CNS in microglia-depleted animals compared to untreated controls, although these T cells nonetheless displayed reduced expression of ‘activation markers’, such as CD69 and CD160 [107]. The recent finding that CD69 is a marker of CNS-resident T cells [250] further confounds this interpretation and more precise analysis will be required to determine the contribution of microglia and/or MDMs to effective CD8^+^ T cell responses in this model.

In addition to decreased APCs in the CNS, the numbers of circulating APCs in the spleen, blood, and pancreatico-duodenal lymph node were also reduced in this model of WNV infection [107]. Thus, reduced peripheral APCs could also have contributed to a sub-optimal systemic CD8^+^ T cell response, although other studies using the same dose and PLX5622 administration route showed no or limited peripheral changes in PLX5622-treated animals (Table 2). Differences in the systemic response to PLX5622 may additionally be due to the age, sex and infection status of mice, or the duration of PLX5622 treatment. Thus, interpretation of studies using PLX5622 to determine the specific functions of microglia must take into account any indirect effects it may cause.

Using alternative experimental systems, including fate-mapping and intravital imaging, Moseman et al. [234] clearly identified a role for microglia in CD8^+^ T cell activation. Microglia were required for the cross-presentation of viral antigen from VSV-infected neurons to CD8^+^ T cells to contain virus and prevent its fatal spread. CD8^+^ T cell recognition of MHC class I (MHC-I) on microglia was crucial for survival, while conditional deletion experiments in this model showed that neuronal MHC-I was not required (Fig. 2) [234].

##### CD4^+^ T cells

Other studies showed a more important role for microglia in CD4^+^ T cell responses. In TMEV infection, PLX5622-driven microglial depletion resulted in reduced CNS infiltration of CD4^+^ T and CD44^+^ CD4^+^ T cells [335], whereas in MHV-infected brains, microglia depletion reduced CD4^+^ and virus-specific, IFN-γ-expressing CD4^+^ T cells [344] (Table 2). In the latter study, the authors argued that the ineffective CD4^+^ T cell response was dependent on reduced microglia/macrophage MHC-II expression and immaturity of infiltrating APC, collectively preventing re-stimulation of CD4^+^ T cells in the CNS (Fig. 2).

##### T regulatory cells

Increased Treg numbers in the CNS resulting from the absence of microglia were also reported in two independent viral encephalitis models (Table 2). Tregs possess several anti-inflammatory mechanisms, including release of interleukin (IL)-10, which dampen the anti-viral immune response and reduce immune-mediated pathology [151]. Thus, their increased infiltration can hamper effective viral clearance. Accordingly, the increased number and percentage of Tregs in the CNS and increased *Il10* mRNA expression in TMEV-infected, PLX5622 microglia-depleted mice suggest a role for microglia in regulating the infiltration of Tregs into the brain [335]. In this study, increased Treg numbers were thought to contribute to an ineffective cytotoxic CD8^+^ T cell response, resulting in decreased viral control and reduced survival in the absence of microglia. Importantly, however, the increase in Treg numbers in this study was not statistically different from the non-depleted, infected control mice. Furthermore, while this study concluded that Treg numbers affected the CD8^+^ T cell response, there was a significant increase in IFN-γ in the CNS and no change in the number of infiltrating CD8^+^ T cells in PLX5622-treated animals, suggesting changes in Treg numbers did not affect a functional T cell response.

Thus, while discrepancies between studies investigating T cell number and their activation in the CNS may suggest a role for microglia in encephalitis caused by particular viruses, many of these studies lack specific evidence for the contribution of microglia to CD4^+^, CD8^+^ or Treg responses. Moreover, the wider effects of PLX5622 on the myeloid and lymphoid compartments [198] make it impossible to assess the specific role of microglia using this molecule alone.

#### The role of microglia in MDM maturation and CNS infiltration

The absence of microglia in virus-infected brains also affects the number of MDM infiltrating into the CNS, suggesting a role for microglia in the recruitment of monocytes from the blood. This phenomenon may be virus-specific, as studies have reported an increase [344], decrease [96, 107] or no change [335] in the number of immigrating MDMs in these diseases (Table 2). Two studies reporting a differential infiltration of MDMs into the virus-infected CNS reported a reduction in the expression of MHC-II or CD86 on these cells (Table 2), arguably supporting a role for microglia in enhancing MDM antigen presentation upon CNS entry. However, the possibility that reduced MDM infiltration is a result of PLX5622 targeting CSF1R, which is highly expressed by these cells, is difficult to exclude.

Despite various groups showing reduced numbers of infiltrating MDMs and cytokine production in the CNS of microglia-depleted mice during infection, these animals were still highly susceptible to lethal encephalitis. This appears inconsistent with the observation that inhibiting monocyte infiltration into the WNV-infected brain reduces immune-mediated pathology and enhances survival [120, 122], while in contrast, reduced MDM infiltration and decreased *Nos2*, *Ifng* and *Tnf* expression in the brains of microglia-depleted WNV-infected mice did not improve survival [107]. These findings suggest that maintaining a microglial network for early defence is as important as reducing subsequent MDM-mediated immunopathology in viral encephalitis.

#### The role of microglial migration and phagocytosis in virus control

The purinergic receptor, P2RY12, was shown to have a role in the control of viral spread in encephalitis caused by PRV, an alphaherpesvirus that infects the brain via retrograde synaptic spread from peripheral neurons [96]. Using advanced imaging techniques in P2RY12-deficient (*P2RY12*^*−/−*^) mice treated with PLX5622 and infected with PRV, this study showed rapid, precise microglial migration to and phagocytosis of virus-infected neurons to reduce CNS spread (Table 2). Microglia/macrophage-mediated engulfment of virus-infected neurons has also been observed in human WNV-infected brains [247] (Fig. 1a). In PRV infection, this process involved microglial migration towards ATP (the ligand for P2RY12) released by infected neurons prior to the appearance of mature virions in the neuronal cytoplasm and before neuronal membranes were compromised. In stark contrast to the transcriptional changes seen in neurodegenerative diseases [174, 179, 191, 214], microglia upregulated P2RY12 by two-fold in this model, demonstrating its importance in CNS infection.

Similarly, during infection with neurotropic VSV, microglia accumulated in the olfactory bulb, forming an ‘innate barrier’ which impeded viral spread to caudal regions of the brain [59]. Consequently, microglial depletion with BLZ945, a different CSF1R inhibitor, resulted in increased VSV load and spread, transforming a sublethal infection into lethal encephalitis. Here, microglial ‘activation’ and accumulation relied on neuron–astrocyte crosstalk. Abrogation of IFN-α/β receptor in neurons and astrocytes, but not microglia, resulted in reduced microglial activation, accumulation, proliferation and enhanced viral spread and mortality. This suggests that IFN-β produced in the olfactory bulbs stimulates IFN-α/β receptor signaling in neurons and astrocytes, indirectly enhancing the microglial anti-viral response.

#### Role of aberrant synaptic pruning in post-viral cognitive dysfunction

Patients recovering from viral encephalitis often show severe neurological sequelae, including deficits in memory, visuospatial and verbal learning, and motor and executive functions [111, 209, 272, 329, 340]. Permanent cognitive dysfunction after infectious encephalitis can occur without neuroinvasion, with cognitive deficits worsening over time [237]. In WNV and Zika virus (ZIKV) encephalitis, microglia have been identified as orchestrators of spatial-learning impairments seen in the recovery phase after viral neuroinvasion [111, 324].

While the phagocytic role of microglia may be protective in the acute phase of viral infection, the same function can become detrimental post infection. This is shown by the inappropriate removal of hippocampal synapses leading to circuitry dysfunction and spatial-learning deficits in the clinical recovery phase from WNV and ZIKV neuroinvasive disease [111, 324]. Both studies show the importance of complement protein C3, the hydrolysed fragment of which, C3a, is recognized by microglial-expressed C3a receptor (C3aR), in the aberrant engulfment of synapses. Discrete immunopathological effects post infection with different flaviviruses were demonstrated by Gaber et al. [109], with WNV infection associated with the enhanced elimination of presynaptic termini and ZIKV infection resulting in neuronal cell death and the enhanced elimination of post-synaptic termini. This study elegantly showed that the persistence of CD8^+^ T cells expressing IFN-γ in the brain parenchyma was required for both microglial activation and the neuronal loss and/or synapse elimination resulting in cognitive dysfunction during the recovery from WNV and ZIKV infection. Accordingly, absence of microglial *Ifngr* prevented the effects of flavivirus-induced hippocampal damage and related clinical deficits [111].

Nonetheless, enhanced microglia-mediated synaptic engulfment during insult, infection or injury may well be a protective mechanism, for instance by preventing excitotoxicity and dampening nonsense signalling activity from damaged or injured neurons [342] or, in the context of neurotropic infection, by limiting trans-synaptic viral spread or aberrant calcium signalling by infected neurons [324]. At the same time, this can also lead to the collateral loss of bystander synapses, a process also observed in CNS pathologies, such as Alzheimer’s Disease (AD) and related dementias [62, 104], and in animal models of Multiple Sclerosis (MS) [10, 21]. While this remains a matter of debate, it has been proposed that C3aR-dependent microglial synaptic engulfment has a role in the functional decline observed in MS [342], AD [152] and aging (Fig. 4). Much like in WNV infection, synapse elimination occurs predominantly at the presynaptic termini and is dependent on the alternative complement cascade (i.e., C3 hydrolysis). Similarly, impaired learning coinciding with synapse and neuronal loss was abolished in aged mice deficient in C3 [285]. Furthermore, IFN-γ-induced C3 expression in an amyloidogenic mouse model reduced plaque load but resulted in increased loss of synapses and cognitive dysfunction [152, 284]. Thus, in various CNS diseases, microglia show conserved pathological mechanisms, which could be targeted for the modulation of disease processes.

### Monocytes in viral encephalitis

The severe neuroinflammatory response associated with viral encephalitis recruits a substantial monocytic infiltrate from the periphery, constituting more than 50% of all recruited cells in some disease models [122, 123]. This significant infiltration is also evident in severe viral encephalitis in humans at postmortem [11, 247, 273] (Fig. 1a). Together with recruited lymphocytes and resident microglia, monocytes carry out inflammatory and anti-viral effector functions necessary for viral clearance [188]. However, this response is not always beneficial and an overexuberant inflammatory response may contribute to fatal encephalitis. A comparison of the differential roles and associated phenotypes (RNA and protein) of microglia compared to MCs in viral encephalitis can be seen in Table 3.

**Table 3:** Functions and phenotypes of microglia and monocytes in viral encephalitis

	Microglia	Monocyte-derived cells
**RNA**	**CD45**^**lo/int**^**CD11b**^**+**^ **microglia** ↑ 'microglia-specific' genes relative to macrophages: *Bmpr1a, Il12b, Gas6, Tnf, Cd74, Ccl12, Csf1, Ly86, Bst2, H2-Aa, H2-Ab1, Ifnb1, Stat1, Tlr2 and Tlr3* (among many others) ↑ reactive ‘microglia-specific’ genes relative to homeostatic microglia: *Itgal, Il12rb1* and *Ccl5* (TMEV infection, dpi 6, bulk RNA-seq [78])	**CD45**^**hi**^**CD11b**^**+**^ **macrophages** ↑ ‘macrophage-specific’ genes relative to microglia: *Gzmb, Il2ra*, *Nos2*, *Oas3*, *Ms4a8a*, *Arg2*, *Trem-1*, *Ly6c2*, *Ccr2*, *Vim*, *Ifi204*, *S100a10* and *Msrb1* (TMEV infection, dpi 6, bulk RNA-seq [78])
**Protein**	**CD45**^**lo/int**^**CD11b**^**+**^ **microglia** ↑ CD45 and MHC-II (TMEV infection, dpi 6, flow cytometry [78])	**CD45**^**hi**^**CD11b**^**+**^ **macrophages** ↑ MHC-II (TMEV infection, dpi 6, flow cytometry [78])
	**CD45**^**int**^**CD11b**^**+**^**Ly6C**^**lo**^ **microglia** ↑ CD45 and Ly6C (Sarafend strain, WNV infection, dpi 7, flow cytometry [123])	**Bead**^**+**^**CD45**^**int**^**CD11b**^**+**^ **macrophages** ↓Ly6C ↑ MHC-II and CD86 (Sarafend strain, WNV infection, dpi 7, flow cytometry [123])
	**CD45**^**+**^**Iba-1**^**+**^**CD68**^**+**^**P2RY12**^**+**^ **microglia** ↑ P2RY12 and CD45 (PRV-infection, dpi 5–7, immunohistochemistry [96])	
	**Four microglia populations in the homeostatic and WNV-infected brain** All differentially express: CD45, CD11b, CX3CR1, F4/80, P2RY12, TMEM119, CD64, MerTK and CD68 **Phenotype 1:** P2RY12hi CD86^+^ **Phenotype 2:** P2RY12lo CD86^+^ **Phenotype 3:** P2RY12hi CD86^−^ **Phenotype 4:** P2RY12lo CD86^−^ Relative to mock-infected mice, the total microglial population in the WNV-infected brain at dpi 7: ↑↑ CD45 and CD64 ↑ CD86 and CD11c ↓↓ CX3CR1, F4/80, TMEM119 and CD68; ↓ P2RY12 (WNV infection at dpi 7, spectral and conventional flow cytometry [298])	
**Neuro-protective roles**	Microglia re-stimulate CD8^+^ T cells [107] and CD4^+^ T cells [344] in the CNS during WNV and MHV infection, respectively, for an effective antiviral T cell response Microglia are required in the earlier stages of MHV infection and their absence is associated with the infiltration of immature antigen-presenting macrophages in the CNS [344] Microglia regulate the infiltration of Tregs and their expression of IL-10 in the CNS of TMEV-infected animals, to prevent the suppression of cytotoxic CD8^+^ T cell activity [335] Microglia rapidly migrate to and phagocytose PRV-infected neurons to prevent viral spread in the brain [96] Microglia accumulate in the olfactory bulb and form an innate immune barrier to prevent the spread of VSV to caudal parts of the brain [59] Microglia prevent the fatal spread of VSV by acquiring viral antigen from VSV-infected neurons and cross-presenting it to CD8^+^ T cells via MHC-I [234]	CCL2- and CCL7-dependent monocyte migration to the brain is required for effective viral clearance and survival in WNV encephalitis (neurotropic WNV) [16] CNS-infiltrating macrophages prevent viremia and enhance survival in WNV encephalitis (neurotropic WNV) [22] CCR2-dependent Ly6C^hi^ monocyte infiltration into the brain is required for effective viral clearance, survival, and anti-viral CD4^+^ and CD8^+^ T cell responses in MHV [54] and WNV encephalitis (neurotropic WNV) [200]
**Neuro-** **toxic** **roles**	IFN-γ signalling in microglia results in neuronal loss and/or synapse elimination during the recovery of WNV and ZIKV infection causing flavivirus-induced hippocampal damage and memory and learning deficits [111]	CNS-infiltrating Ly6C^hi^ monocytes and macrophages contribute to seizure incidence, seizure severity, memory deficits or hippocampal neuron damage in TMEV-induced encephalitis [70, 155, 334] IL-6-producing CD45^hi^CD11b^+^ myeloid cells correlate with seizure development in TMEV encephalitis [70, 79] Ly6C^+^ MDM trafficking into the brain correlates with morality in WNV encephalitis (Sarafend strain) [122, 123] Infiltration of NO-producing Ly6C^+^ MDM into the CNS correlates with mortality in lethal WNV encephalitis (Sarafend strain) [122]

#### Monocyte-mediated viral clearance contributes to secondary tissue damage

Upon entry into the infected brain, MDMs can present viral antigen and support CD4^+^ T cell-mediated viral clearance or contribute to the killing of infected cells through release of inflammatory mediators, such as NO and IL-6 [16, 22, 54, 200] (Fig. 2). While these responses enhance pathogen clearance, they can also contribute to substantial neurodegeneration [70, 120, 122, 123, 155, 334]. During WNV infection in the mouse, for instance, the infiltration of Ly6C^hi^ monocytes into the infected brain coincides with the onset of fatal encephalitis [122, 123]. These inflammatory monocytes are recruited to the CNS in a C–C Motif Chemokine Ligand 2 (CCL2)- and very late antigen-4 (VLA-4)-dependent manner and exacerbate CNS injury through sustained production of NO [122, 123]. Inhibiting monocyte infiltration into the brain with anti-CCL2 and anti-VLA-4 antibody blockade or inhibiting their inflammatory activation by inhibition of *Nos2* with aminoguanidine hemisulphate significantly increases survival in infected mice without altering viral titre [122, 123], strongly supporting the notion that monocytes and MDMs mediate inflammatory damage in WNV infection.

The detrimental role of monocytes in the context of viral encephalitis is likely confined to the CNS parenchyma [122], as these cells play an essential and beneficial role in controlling WNV infection in the periphery prior to their infiltration into the CNS [73], although they also contribute to significant local tissue damage in the periphery in alphavirus infection [363]. This is consistent with studies showing that the reduction of monocytes into the CNS using CCR2-, CCL2-, and CCL7-deficient mice or in vivo clodronate liposome administration increased viral burden when virus was peripherally inoculated [16, 22, 200]. Upon entry into the infected brain, monocytes express MHC-II, CD80 and CD86 and have the capacity to present antigen and stimulate the proliferation of activated T cells [122] (Fig. 2), together supporting their role in inducing anti-viral T cell responses. It is possible that a detrimental or beneficial response of monocyte-derived cells in WNV encephalitis depends on the virus strain, dose, and route of inoculation. However, considering their relatively late arrival into the CNS parenchyma [122], whether these cells contribute to WNV clearance in the brain is still unclear.

#### Potential role of monocytes in seizure development

The recruitment of monocytes into the CNS following infection with TMEV is also thought to significantly contribute to hippocampal neurodegeneration [155] and seizure development during viral encephalitis [70, 334]. This pathological role is emphasized by studies demonstrating that the in vivo depletion of myeloid cells with anti-Gr-1 antibody or with anti-inflammatory agents, wogonin and minocycline, preserved cognitive functions and reduced seizure incidence [70, 155, 334]. Although these studies attributed ameliorated disease signs to reduced monocyte trafficking into the brain, the findings are potentially confounded by non-specific depletion methods; thus, the participation of other cell types cannot be excluded. Using a different monocyte-depletion method with clodronate liposomes (which deplete phagocytes in the bloodstream, spleen, and BM [319–321]), seizure severity was improved but there was no amelioration in hippocampal neurodegeneration [334], suggesting the observed contribution of monocytes to pathology is dependent on the depletion method. Altogether, whether seizures originate innately in embryologically primed, infected neurons or exogenously from parenchymal or other infiltrating cell stimuli remains unclear [70, 120, 122, 123, 155, 334] and deciphering the functional roles of monocytes in viral encephalitis-induced seizures will require more precise investigation.

## Ischemic injury and repair

Stroke, which can be generally divided into haemorrhagic stroke and ischemic stroke, accounts for approximately 10% of all deaths and 5% of all disability-adjusted life years worldwide [239]. Ischemic strokes are the most prevalent type of stroke [176], and they are caused by arterial occlusion, which is most commonly caused by large vessel atherosclerosis and plaque rupture, cardioembolism, and small vessel disease (typically linked to hypertension) [176, 330]. In general, however, ischemic stroke is a clinically heterogeneous condition determined by the degree, duration, and location of ischemia, as well as age, sex, and multi-medication comorbidities [290]. Although rodent models may reproduce the consequences of an ischemic insult, they often fail to recreate the complex pathophysiology that leads to an endogenous stroke [208].

### Relevance of murine models to human ischemic stroke

Animal models of ischemic stroke carry significant limitations in replicating the aetiology and time course of human disease. Many researchers have attempted to model disease pathogenesis in young, healthy male rodents [51, 83, 208], despite the fact that ischemic stroke is most prevalent in older individuals [24, 95] and is strongly linked to systemic diseases, such as hypertension, hypercholesterolemia, obesity, and diabetes mellitus [14]. With the exception of thromboembolic clot models [290], these models also fail to replicate the delayed spontaneous reperfusion reported in 17% of human thromboembolic strokes [175]. While the sequence of events following cerebral ischemia is similar in humans and rodents, the kinetics of this response also vary substantially [154]. This impacts the therapeutic window for reversible ischemic damage [154], as well as the functional recovery period, which can span years in humans [47]. These discrepancies between stroke animal research and clinical practice have been identified as a leading cause of translational failures [28, 158], prompting significant reform in study design and experimental model selection in the field [33, 100, 157, 207].

These limitations have recently been addressed by either inducing stroke in aged animals with pre-existing comorbidities (e.g., diabetes, hyperlipidemia, obesity, infection), reviewed in [217], or using animal models with risk factors that eventually result in spontaneous stroke (e.g., spontaneous hypertensive rat and stroke-prone spontaneously hypertensive rat) [92, 245]. Although these models more accurately mimic human ischemic stroke [290], they nonetheless recreate only individual aspects of this clinically heterogeneous disease [208]. Therefore, investigations attempting to generalize findings from isolated experimental models of ischemic stroke should be interpreted with extreme caution.

Nonetheless, animal models have been invaluable in understanding evolving brain injury following an ischemic insult [84]. Within minutes of blood flow interruption (15–20% below baseline), irreversible cell death ensues, resulting in the formation of an infarct core [85]. The penumbra, or non-functional tissue around the infarct core, may recover; however, if blood flow is not restored, this "at-risk" tissue will be incorporated into the infarct core [13, 305]. Infarcts often continue to expand even after blood flow is restored to the occluded region, in the process of ischemia–reperfusion injury [244].

The sequence of events following an ischemic injury and involvement of resident and peripheral immune cells is remarkably similar between human disease and its experimental models. Examination of post-mortem human stroke lesions demonstrates that inflammation begins early after ischemic insult [223] and the surrounding penumbral tissue is rapidly surrounded by ‘activated’ microglia [90]. This inflammatory response is associated with the release of damage-associated molecular patterns (DAMPs) from injured neurons, BBB breakdown, and infiltration of peripheral immune cells, including monocytes and neutrophils, into the ipsilateral hemisphere [90], with macrophages, mononuclear cells, and perivascular cuffing present in 75%, 44%, and 42% of human brains with ischemic infarcts, respectively [223]. Although infiltrating immune cells may act in concert with resident cells to initiate debris clearance and tissue repair processes [369], acute inflammation may persist and transform into chronic, non-resolving inflammation. Inflammatory mononuclear cells and macrophages may persist for up to 53 years following the initial ischemic insult [223] and are often present in chronic lesions (Fig. 1b). Aberrant acute inflammation may also contribute to secondary injury, aggravating tissue damage and increasing lesion volume [160]. As a result, the neuroinflammatory response to ischemic injury is a critical determinant of brain damage and neurological recovery following a stroke. Despite variations in the kinetics the inflammatory response underlying this process [171], animal models show cross-species parallels in the immune response to ischemic injury.

### Microglia in stroke

#### Microglial modulation of dysfunctional CNS cellular responses in stroke

**Fig. 3:**
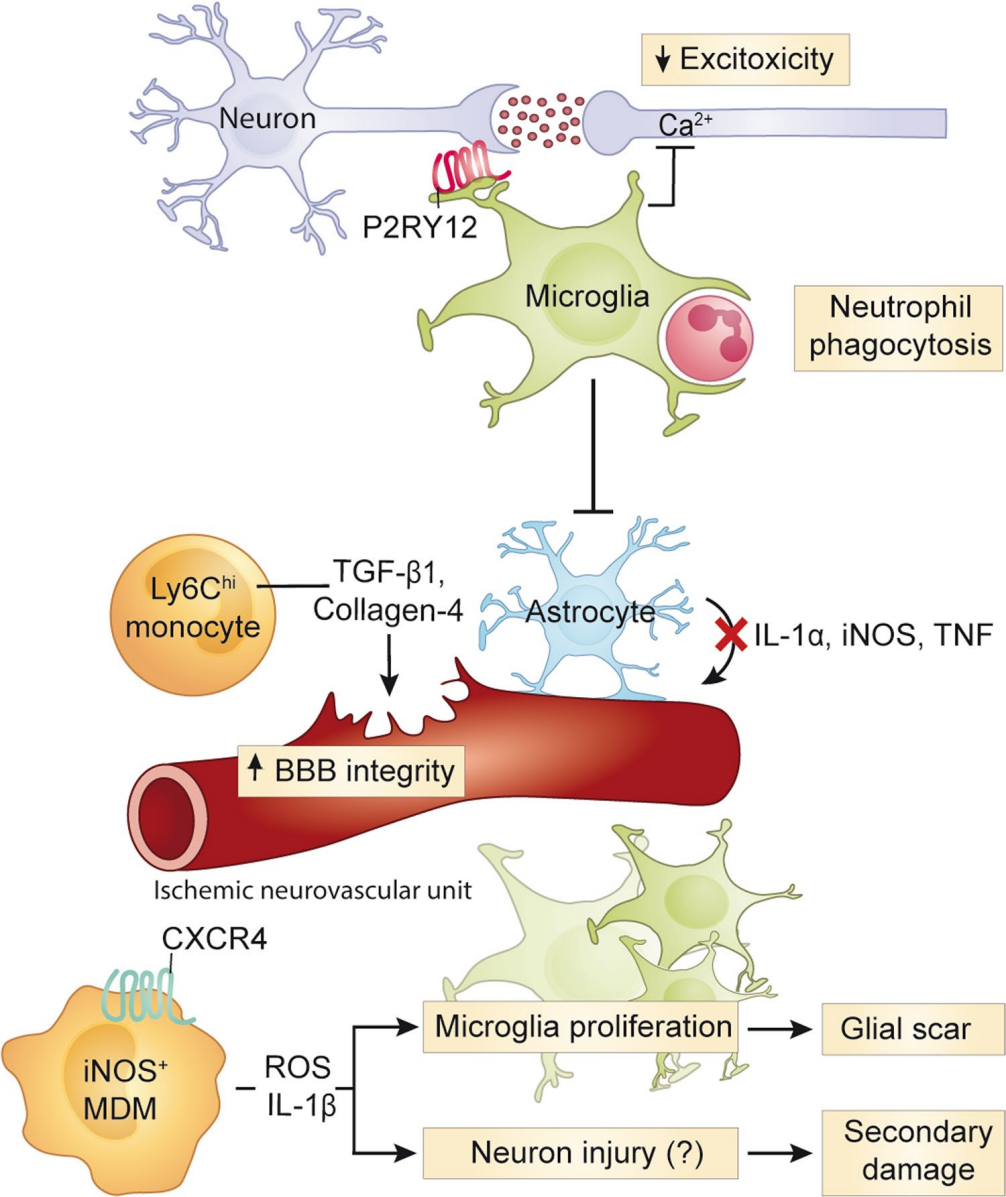
Roles of monocytes and microglia in ischemic stroke. In stroke, microglia migrate towards neurons with high intracellular calcium levels to reduce excitotoxicity and neuronal damage. Microglia also prevent bystander tissue damage by inhibiting reactive astrocytes and phagocytosing infiltrating neutrophils. CXCR4^+^ MDM are recruited to the site of injury, where they produce microglia-activating mediators, IL-1β and ROS, and stimulate microglia proliferation and the glial scar formation. These inflammatory mediators may also injure neurons and contribute to secondary damage. CNS-infiltrating Ly6C^hi^ monocytes may also help repair the damaged BBB via the production of collagen-4 and TGF-β1. BBB, blood–brain barrier; CXCR4, CXC chemokine receptor type 4; CXCL12, CXC Motif Chemokine Ligand 12; IL, interleukin; MDM, monocyte-derived macrophage; ROS, reactive oxygen species; TGF-β1, transforming growth factor beta 1

The roles of microglia in the early stages of ischemic injury have been largely elucidated by experimental models of ischemic stroke induced by middle cerebral artery occlusion (MCAO), which generates reproducible infarcts in the middle cerebral artery region and allows reperfusion after removal of the occluding filament (*i.e.,* transient MCAO). Although blood flow restoration in transient MCAO does not accurately depict spontaneous reperfusion in human stroke, it is a better representation of mechanical thrombectomy or thrombolysis in the human than endogenous stroke [208]. In models of transient or permanent cerebral ischemia induced by MCAO, microglial depletion increased infarct size and worsened disease outcome, suggesting that microglia play a beneficial role in the early phases of stroke. Studies have suggested that this protective role may include direct cell interactions with surrounding parenchymal cells, resulting in the containment and/or prevention of dysregulated neuron [117], neutrophil [248] and astrocyte [172] responses that occur following ischemic insult (Fig. 3).

Experimental microglia depletion in animals has provided insight into the immune processes that protect against secondary injury, which may also underlie human disease. Two MCAO studies found an increase in neuronal death and infarct size after microglia were depleted by continuous oral injection of PLX3397 starting three weeks before stroke induction [117, 172] (Table 4). Increased tissue damage was linked to increased intraneural calcium levels and excitotoxic injury in one study, suggesting that microglia may play a role in protecting neurons from injury associated with high intracellular calcium levels [117] (Fig. 3). Importantly, the increased infarct damage did not occur if microglia were allowed to repopulate by removal of PLX3397 before stroke induction, thus emphasising their protective role in stroke. Microglia rapidly migrated to infarct sites and formed contact interactions with neurons with high intracellular calcium levels, likely reducing excitotoxic damage. At the interface between microglia and neurons, the purinergic receptor P2RY12 was highly expressed. P2RY12 is considered a homeostatic microglia marker because of its downregulation in neurogenerative and neuroinflammatory models, including amyotrophic lateral sclerosis, AD (Table 5) and MS (Table 6) [174, 179, 191, 214], which have been more extensively investigated than encephalitis (Table 3) and stroke (Table 4). Considering P2RY12 is upregulated by microglia in stroke [117] and is required for the control of viral spread in PRV encephalitis [96], it may not be a general homeostatic microglia marker as currently proposed. The transcriptomes and proteomes of microglia and MC in viral encephalitis, stroke, AD and MS are listed in Tables 3, 4, 5, 6, respectively.

**Table 4:** Functions and phenotypes of microglia and monocytes in stroke

	Microglia	Monocyte-derived cells
**RNA**	***Cxcr4*-GFP**^**−**^**CD11b**^**+**^**CD45**^**lo**^ **microglia** ↑ *Siglech*, *Cx3cr1* and *C1qb* (‘microglia-specific’ genes) ↑ *Plxna4*, *P2ry12* and *P2ry13* (cell surface receptor) ↑ *Gpr34*, *Gpr56*, *Gpr84* and *Adrb2* (heptahelical receptors) ↑ *Tlr3* and *Tmem173* (pattern recognition receptors) ↑ *Mki67* (proliferation marker) (PT, bulk RNA-seq [343])	***Cxcr4-*GFP**^**+**^**CD45**^**hi**^**CD11b**^**+**^**Ly6C**^**hi**^ **MDM** ↑ *Siglech, Cx3cr1* and *C1q* (‘microglia-specific’ genes) ↑ *Cybb* and the Src kinase *Fgr* (super-oxide-generating) ↑ *Tlr8*, *Ifi202*, *Clec7a*, *Clec4d* and *Oas2* (pattern recognition molecules) ↑ *C3*, *Cfb* and *Cfp* (complement system components) ↑ *H2-Aa*, *H2-Ab1* and *CD74* (antigen processing and presentation via MHC-II) ↑ *Irf7*, *Ifi200b*, *Ifi202, Ifitm2*, *Ifitm3*, *Oas2, Oasl2*, *Rsad2*, *Trim25* and *Tlr8* (interferon-related genes) ↑ *Ccr1*, *Ccr2*, *Cxcr4, Plxna1*, *Plxnc1*, *Plxnd1*, *Adgre5*, *Gpr35*, *Gpr65* and *Gpr132* (cell surface receptors) ↑ *Thbs1*, *Emp1*, *Ifi207* and *Dab* (Other genes) (PT, bulk RNA-seq [343])
**Protein**	**CD45**^**int**^**CD11b**^**+**^**Ly6C**^**−**^ **microglia** (tMCAO, flow cytometry [269])	**CD45**^**hi**^**CD11b**^**+**^**Ly6C**^**+**^**Ly6G**^**−**^ **monocytes** (tMCAO, flow cytometry [269])
	**CD45**^**lo**^**CD11b**^**+**^**Ly6C**^**−**^ **microglia** (PT and tMCAO, flow cytometry [343])	***Cxcr4*-GFP**^**+**^**CD45**^**hi**^**CD11b**^**+**^**Ly6C**^hi^ **Ly6C**^**hi**^ **monocytes/MDM** (PT and tMCAO, flow cytometry [343])
	***Cxcr4*-GFP**^**+**^**Iba1**^**+**^**TMEM119**^**+**^ **microglia** (tMCAO, immunohistochemistry [343])	**CD45**^**hi**^**CD11b**^**+**^**Ly6C**^**lo**^**CX3CR1**^**int**^ **MDM** ↑ CX3CR1, CD206 and Dectin-1 ↓ Ly6C (tMCAO, flow cytometry [339])
	**CD45**^**+**^**CD11b**^**+**^**TMEM119**^**+**^ **microglia** (tMCAO, flow cytometry [168])	
**Neuro-protective roles**	Microglia sense alterations in neuronal calcium levels, migrating to neurons in infarct regions high in calcium and forming contact interactions to reduce excitotoxic injury [117]	Ly6C^hi^ monocyte infiltration correlated with the expression of anti-inflammatory genes *TFG-β, CD163* and *Ym1* and are required for long-term functional recovery from stroke [339] Depletion of Ly6C^hi^CCR2^+^ monocytes worsened functional outcomes and increased infarct volume 24 h post-stroke [60] Ly6C^hi^ monocyte infiltration prevents hemorrhagic infarct transformation and correlates with tissue expression of *collagen-4, TGF-β1* and *thrombospondin-1* genes, implicating a role for BBB maintenance following ischemic injury [127]
	Microglia in the periphery of infarcts phagocytose infiltrating neutrophils to prevent their accumulation. Neutrophils can induce bystander tissue damage which enhances ischemic lesion size and brain injury [248]	
	Microglia inhibit dysfunctional astrocyte responses by reducing their expression of pro-inflammatory mediators [172]	
**Neuro-toxic roles**	Microglia depletion in diabetic animals with MCAO decreases brain injury and improves survival and cognition, which was associated with preserved working memory, increased myelination in the white matter and reduced inflammatory macrophage infiltration into the CNS [168]	Ly6C^hi^ monocyte infiltration exacerbates infarct volume at 24 h and five days post-stroke [81, 192], and contributes to early motor deficits in the first three days post-intracerebral hemorrhage [143]

**Table 5:** Functions and phenotypes of microglia in Alzheimer's disease

**RNA**	**Three microglia phenotypes.** All express: *Hexb* and *Cst3* **‘Homeostatic’ phenotype:** *Cx3cr1, P2ry12, Tmem119, Hexb, Cst3, Cx3cr1, Ctsd, Csf1r, Ctss, Sparc, Tmsb4x, P2ry12, P2ry13, C1qa* and *C1qb* **TREM-2-independent, ‘intermediate state’ microglia phenotype:** ↑ *Tyrobp, Apoe, B2m, Ctsd, Ctsb, Fth1* and *Lyz2* *↓Cx3cr1, P2ry12, P2ry13* and *Tmem119* **TREM-2 dependent, damage-associated microglia (DAM) phenotype:** ↑↑ *Tyrobp, Apoe, B2m, Ctsd, Ctsb, Fth1* and *Lyz2* ↑*Cst7, Lpl, Trem2, Axl, Cstsl, Cd9, Csf1, Ccl6, Itgax, Clec7a, Lilrb4* and *Timp2* *↓↓ Cx3cr1, P2ry12, P2ry13* and *Tmem119* (5xFAD at 3 and 8 months, single-cell RNA-seq [179])
	**Microglia neurodegenerative phenotype (MGnD)** *↓↓ P2ry12*, *Tmem119*, *Gpr34*, *Jun*, *Olfml3*, *Csf1r*, *Hexb*, *Mertk*, *Rhob*, *Cx3cr1*, *Tgfbr1*, *Tgfb1, Mef2a*, *Mafb*, *Jun*, *Sall1* and *Egr1 (‘homeostatic’ genes)* ↑ *Spp1*, *Itgax*, *Axl*, *Lilrb4*, *Clec7a*, *Ccl2*, *Csf1* and *Apoe* (‘inflammatory’ genes) (APP-PS1 at 7, 10 and 17 months. MGnD is also seen in ALS (SOD1^G93A^ mice) and in MS (acute EAE), single-cell RNA-seq [191])
**Protein**	**Three microglia phenotypes:** **Phenotype 1:** Clec7a^−^P2ry12^+^ (not associated with Aβ plaques) **Phenotype 2:** Clec7a^lo^P2ry12^lo^ (in close proximity to Aβ plaques) **Phenotype 3:** Clec7a^+^P2ry12^–^ Transition from Clec7a^−^ to Clec7a^int^ to Clec7a^hi^ correlated with increased mRNA expression of *Apoe* and suppression of homeostatic molecules (APP-PS1, immunohistochemistry [191])
	**Three FCRLS**^**+**^**CD11b**^**+**^ **microglia phenotypes:** **Phenotype 1: **Clec7a^−^ **Phenotype 2: **Clec7a^int^ **Phenotype 3: **Clec7a^+^ (APP-PS1, flow cytometry [191])
	**Iba-1**^**+**^**CD11c**^**+**^**TIMP2**^**+**^ **microglia** Co-localized with Aβ^+^, *Csf1* and *Lpl* (5xFAD, immunohistochemistry & smFISH^a^ [179])
**Neuro-** **protective** **roles**	Microglia encircle Aβ plaques to prevent further growth and dissemination into the parenchyma [63], reducing damage to local neurites [295] Microglia may contribute to the phagocytic and enzymatic clearance of the Aβ plaque deposits [82] TREM2 expressed by damage-associated microglia is thought to directly recognise Aβ to enhance engulfment and lysosomal degradation of the protein [338, 357, 366] TREM2-dependent microglia functions are required to prevent seeding of plaques earlier in AD, whilst later on enhance the consolidation of Aβ into highly compact plaques [222]
**Neuro-** **toxic** **roles**	Microglia cause synapse and neuronal damage, injury, or loss, resulting in memory loss and cognitive decline [152, 284, 291, 294–296] Microglia contribute to the formation and growth of Aβ plaques [294, 295] Microglial secretion of tau-laden exosomes help seed and spread tau aggregates throughout the CNS [12, 61]

^a^Single molecule fluorescence in situ hybridization

**Table 6:** Functions and phenotypes of microglia and monocytes in models of Multiple Sclerosis

	Microglia	Monocyte-derived cells
**RNA**	**FCRLS**^**+**^ **CD11b**^**+**^ **microglia** All express: ↓↓ *P2ry12*, *Tmem119*, *Tgfbr1*, *Mafb*, *Mef2a*, *Sall1* and *Egr1* (homeostatic genes) ↑ *Apoe* (pro-inflammatory molecule) Downregulated/upregulated genes in acute phase of EAE were restored to homeostatic levels during recovery phase (acute EAE, single-cell RNA-seq [191])	**Ly6C**^**hi**^**CD11c**^**+**^**MHC-II**^**+**^**CD11b**^**+**^**CD45**^**hi**^ **MoDC** All express: GM-CSF-dependent gene signature: ↑ *CCL6, CCL17, CCL24* and *Tnfrsf9* (co-stimulatory molecules) ↑ *Mfge8*, *Cd1d1*, *Pld1*, *Scarb1*, *Clec7a* and *Anxa1* (phagocytosis-associated genes) ↑ *Asc, NLRP3,* and *Pycard* (inflammasome-associated genes) (peak EAE, next-generation sequencing [67])
	**Four disease-associated microglia (daMG) phenotypes.** All express: *Bhlhe41*^*lo*^*, Gpr34*^*lo*^*, Hexb, Olfml3, P2ry13, Sall1*^*lo*^*, Serpine2*^*lo*^*, Siglech*^*lo*^*, Sparc*^+^*, Maf*^*lo*^*, Slc2a5*^*lo*^*, Ccl2*^*hi*^*, Cxcl10*^*hi*^*, Ly86*^*hi*^*, Mki67*^+^*, Selplg*^*lo*^*, Cx3cr1*^*lo*^*, Fcr1*^*lo*^*, Csfr, Csf1, C1qc, C1qb* and *C1qa* **daMG 1:** *Cd83*^*hi*^, *Ctsd*^*hi*^, *Cd8*^*hi*^, *P2ry12*^+^ and *TMEM119*^+^ **daMG 2:** *Cd83*^*hi*^, *Ctsd*^*hi*^, *Cd8*^*hi*^, *P2ry12*^+^ and *TMEM119*^+^ (not found in lesions) **daMG 3:** *Ctsb*^*hi*^, *Apoe*^*hi*^, *B2m*^*hi*^, *Cst7*^*hi*^, *Mpeg1*^*hi*^, *CD74*^*hi*^, *Cxcl10*^*lo*^, *P2ry12*^*lo*^ and *TMEM119*^*lo*^ (more likely to make contact with T cells) **daMG 4:** *Itm2b*^*hi*^, *Ctss*^*hi*^, *Ccl5*^*hi*^, *Naaa*^*hi*^, *CD74*^*lo*^, *P2ry12*^*lo*^ and *TMEM119*^*lo*^ (daMG 3 and 4 have the highest proliferative capacity) (peak EAE, single-cell RNA-seq [174])	**Four monocyte-derived cell populations in the parenchyma and perivascular space.** All express: *Ly6c2, Ccr2, Cd44* and *Fcgr1* **Ly6C**^**hi**^ **monocytes:** *Fn1* **MertK**^**+**^ **MDM subset 1:** *Fn1* and *Mertk* **MerTK**^**+**^ **MDM subset 2 and 3:** *Fn1, Mertk, Mrc1* and *Ms4a7* **MoDC: ***Fn1*, *Kmo* and *Zbtb46* (peak EAE, single-cell RNA-seq [174])
	**Three microglia (Mg) populations.** All express: *P2ry12*, *Tmem119*, *Cx3cr1*, *Hexb* and *Olfml3* Top 10 DEG^a^ and top GO^b^ terms associated with each population: **Mg III:** *Cd74, Ifi27l2a, Cst7, Fcgr4, Lgals3bp, Cxcl10, Iigp1, H2-K1, H2-D1* and *Fgl2* (antigen processing and presentation) **Mg IV:** *Stmn1, Top2a, Hmgb2, Ube2c, 2810417H13Rik, Birc5, Cks1b, Spp1, Ifi27l2a* and *Ccl12* (cell division) **Mg V:** *AA467197, Lgals3, Lyz2, Arg1, AW112010, Plac8, Cxcl2, Ccl5, Il1b* and *Tgfbi* (ROS metabolic process) Also expressed the ‘oxidative stress’ signature *Cybb, Ncf2, Ncf4* and *Gpx1* (onset EAE, Toxic single-cell RNA-seq [224])	**Seven monocyte populations:** All express: **Ly6C**^**hi**^ **monocytes:** *Ly6c2, Sell* and *Ccr2* **Ly6C**^**lo**^ **monocytes:** *Nr4a1* and *Pparg* **Ifit2**^**+**^ **monocytes:** *Ifit1, Ifit2, Ifit3, Usp18* and *Irf7* **Arg1**^**+**^ **macrophages:** *Arg1, Apoc2* and *C1qb* **Nos2**^**+**^ **macrophages:** *Nos2, Gpnmb, Arg1*and *Fabp5* **Saa3**^**+**^ **monocytes:** *Saa3, Plac8* and *Gbp2* **Cxcl10**^**+**^ **monocytes:** *Cxcl9, Cxcl10* and *Il1b* (peak and chronic EAE, MARS-seq [124])
		**Seven monocyte/macrophage (Mp) populations:** All express: Top 10 DEG^a^ and top GO^b^ terms associated with each populationL **Mp I: ***Clec4n, Inhba, Cd9, Ccl6, Clec7a, Cfb, Bcl2a1d, Il1a, Nos2* and *Arg1* (ROS metabolic process) **Mp II:** *Cd81, Sparc, Ccl12, C1qa, C1qc, Hexb, Cx3cr1, Ly86, Olfml3* and *Cd63* (inflammatory response) **Mp III:** *Apoe, C1qc, C1qa, C1qb, Apoc2, Trem2, Ccl5, Ms4a7, H2-Eb1* and *H2-Aa* (antigen processing and presentation via MHC-II) **Mp IV:** *Apoe, C1qa, Ms4a7, C1qb, C1qc, Lgmn, Cx3cr1, Ccl12, Ly86* and *Trem2* (lipid catabolic process) **Mp V:** *Plac8, Isg15, Gbp2, Ms4a4c, Ifitm1, Ccr2, Tgm2, Actb, Fgl2* and *Ifit3 (r*esponse to interferon-β) **Mp VI: ***Cxcl10, Ifitm6, S100a4, Lrg1, Ifi203, Gm9733, Ifitm2, Tspan13, Wfdc17* and *Tmem176b (*cytokine production) **Mp VII:** *S100a9, S100a8, Ngp, Camp, Retnlg, Lcn2, 1100001G20Rik, Ltf, Ifitm6* and *Pglyrp1 (*leukocyte migration) (onset EAE, Toxic single-cell RNA-seq [224])
**Protein**	**Three microglia populations.** All express: CD45^+^CD11b^+^CD317^+^CD88^+^MHCI^+^MerTK^+^4D4^+^FCRLS^+^ **Population A:** MHC-II^−^CD39^lo^CD86^−^ (also found in homeostatic brain) **Population B:** MHC-II^−^CD39^hi^CD86^+^CD80^+^TIM4^+^ CCR5^+^CCR4^+^ CD206^lo^TREM2^lo^ (also found in homeostatic brain) **Population C:** MHCII^+^CD39^hi^CD86^+^CD80^+^Axl^+^ TIM4^+^PD-L1^+^ CD11c^+^CCR5^+^CD206^lo^TREM2^lo^ (arises during EAE & Huntington’s Disease) (onset and peak EAE, CyTOF [4])	**Five monocyte-derived cell subsets.** All express: CD45^+^CD11b^+^Ly6C^+^ **Subset D:** PD-L1^+^MHC-II^+^Axl^hi^MerTK^int^TREM2^int^ CD86^hi^CD80^hi^CD206^lo^CD39^hi^CD38^hi^ **Subset E:** PD-L1^+^MHCII^+^Axl^hi^MerTK^int^ TREM2^int^CD86^hi^CD80^hi^CD206^lo^CD39^hi^CD38^hi^ **Subset F:** PD-L1^−^CD88^−^IL-17R^−^Axl^−^MerTK^−^TREM2^−^CD86^int^CD80^−^CD206^−^CD39^int^CD38^lo^ **Subset G:** PD-L1^−^CD88^+^IL-17R^−^Axl^−^MerTK^−^ TREM2^lo^CD86^lo^CD80^lo^CD206^−^CD39^lo^CD38^int^ **Subset H:** PD-L1^−^CD88^+^IL-17R^+^Axl^lo^MerTK^lo^ TREM2^lo^CD86^int^CD80^lo^CD206^lo^CD39^int^CD38^int^ (pre-symptomatic, peak, recovery and chronic EAE, CyTOF [4])
	**Three microglia populations.** All express: CD45^+^CD11b^+^CD317^+^CD88^+^MHCI^+^MerTK^+^4D4^+^FCRLS^+^ **Population A:** MHC-II^−^CD39^lo^CD86^−^ **Population B: **MHC-II^−^CD39^hi^CD86^+^CD80^+^Ax1^+^TIM4^+^ CCR5^+^CD206^lo^TREM2^lo^ **Population C:** MHC-II^+^CD39^hi^CD86^+^CD80^+^Axl^+^ TIM4^+^PD-L1^+^ CD11c^+^CCR5^+^CD206^lo^TREM2^lo^ (chronic and recovered EAE, CyTOF [4])	**Two monocyte-derived cell (MC) subsets** All express: **MerTK**^**+**^ **MC:** MerTK^+^CD64^+^Ly6C^+^CD44^+^ **CD209**^**+**^ **MC:** CD209^+^CD64^+^Ly6C^+^CD44^−^ (peak EAE, flow cytometry [174])
	**Four disease-associated microglia (daMG) populations.** All express: Iba-1^+^SPARC^+^Ly86^+^CD162^+^ **daMG1:** MD-1^−^P2RY12^+^TMEM119^+^ (not localized in lesions) **daMG2:** MD-1^+^P2RY12^lo^TMEM119^lo^CD74^+^ **daMG3: **MD-1^+^P2RY12^lo^TMEM119^lo^CXCL10^+^ **daMG4:** MD-1^+^P2RY12^lo^TMEM119^lo^CCL5^+^ –daMG 2–4 were found localized in lesions (peak EAE, immunohistochemistry [174])	**CD45**^**hi**^**CD11b**^**+**^**F4/80**^**+**^ **macrophages** All express: Upregulation of MHC class II (JHMV-induced demyelination, flow cytometry [277])
**Neuro-protective roles**	Microglia prevent dysfunctional pro-inflammatory astrocyte responses in toxin-mediated demyelination enabling an inflammatory/regenerative switch required for the initiation of remyelination [352] CSFR signalling causes the expansion of a CD11c + microglia population, corresponding with suppression of disease progression and disease severity, and the reduction in demyelination and the loss of oligodendrocytes [347] Stimulation of P2X4R on microglia enhances an acidic shift in lysosomes which increases microglial phagocytic capacity to promote myelin clearance and remyelination [362] Blocking TREM-2 or mice deficient in TREM-2, revealed a failure of microglia to upregulate genes required for lipid metabolism and phagocytosis, and resulted in the exacerbation of EAE [254, 255] Microglia are protective in the development of secondary progressive MS by suppressing T cell activation and reducing neuronal degeneration [309]	NO^+^ and Arg1^+^ monocyte-derived cells may represent CD11b^+^Ly6C^hi^Ly6G^−^F4/80^+^CD93^+^ cells, a subset of myeloid-derived suppressor cells, capable of suppressing CD4^+^ and CD8^+^ T cells through the production of NO in culture [367] Adoptively transferred monocytes treated with the MS drug, glatiramer acetate, reversed EAE paralysis by inducing MHC-II-restricted Treg and T helper 2 cells in an antigen-independent manner [341]
**Neuro-** **toxic** **roles**	Microglia depleted animals had more mature oligodendrocytes, suggesting that microglia play a neurotoxic role in the acute phase of EAE by preventing oligodendrocyte progenitor cell maturation and remyelination [243] Peli deletion (*i.e.,* *Peli*^*−/−*^ mice) [206] and ablation of Tak1 in long-lived CX3CR1^+^ cells [129] (which includes microglia) attenuated EAE pathology. Peli and TAK1 are involved in production of pro-inflammatory chemokines and cytokines. Thus, microglial proinflammatory mediator production is detrimental in the development of EAE Microglial engulfment of presynaptic termini contributes to synapse loss [342] Targeting microglia and MDMs expressing an ‘oxidative stress signature’ with acivicin decreased axonal damage, demyelination and the infiltration of immune cells into the CNS in EAE, whilst reducing microglia activation and enhancing neuronal survival in microglia-mediated demyelination [224], suggesting that a subpopulation of microglia are neurotoxic in EAE	CCR2 [98, 137, 165, 230] or CD49e [4]-dependent Ly6C^hi^ monocyte infiltration into the spinal cord is necessary for EAE induction [4, 98, 137, 165, 230] and exacerbates disease severity [4] MDM initiate myelin destruction at the Nodes of Ranvier [355] CD11c^+^CCR2^+^ MoDC may stimulate myelin-reactive T cells, as selective deletion of MHC-II on both peripheral and CNS-resident CD11c + myeloid cells, but not CNS-resident myeloid cells alone, prevented EAE induction, and CCR2^+^ peripheral myeloid cells preferentially show long-lasting interactions with autoreactive CD2^+^ T cells [174] GM-CSF-stimulated monocyte-derived cells contribute to EAE induction [67], and clinical disease severity [67], and participate in de-myelination [67, 297] via the production of IL-1β and ROS [297]

^a^Differentially expressed genes

^b^Gene ontology

In a different MCAO study, enhanced brain injury in the absence of microglia was associated with an increased production of pro-inflammatory mediators by astrocytes (IL-1α, IL-1β, iNOS, IL-6 and TNF), implicating a role for microglia in inhibiting dysfunctional astrocyte responses [172] (Fig. 3 and Table 4). Moreover, after PLX3397-mediated microglia depletion, MCAO in either *Rag2*^*–/–*^*γc*^*–/–*^ mice (which lack T, B, Natural Killer and Natural Killer T cells), or in mice treated with Bindarit (which inhibits CCL2-mediated monocyte infiltration) resulted in enhanced infarct injury compared to mice without PLX3397, emphasizing that the absence of microglia, rather than the presence of a particular infiltrating immune cell type, exacerbates brain injury [172].

Besides the described protective interactions with astrocytes and neurons, microglia also exert beneficial functions in the brain by phagocytosing infiltrating neutrophils, thereby preventing their pathogenic accumulation and subsequent damage to the surrounding tissues and vasculature [248]. Indeed, inhibition or genetic deletion of neutrophil elastase, blocking neutrophil recruitment and/or infiltration, or inhibiting neutrophil-derived matrix metalloproteinase-9, all reduce lesion progression [170, 218, 248, 301]. Accordingly, depletion of microglia before stroke induction increased neutrophil numbers in the CNS and exacerbated tissue damage [248]. Thus, microglia crucially minimise pathogenic neutrophil accumulation to limit ongoing secondary oxidative damage of surrounding re-perfused vasculature and brain tissue [248] (Fig. 3 and Table 4).

However, the beneficial roles played by microglia after ischemic stroke are mostly based on studies in young mice, which do not reflect changes in the aging immune system that would occur in the majority of stroke patients. Studies investigating these age-related changes in the immune responses to MCAO have demonstrated that, although older and young mice have a similar inflammatory response profile, the magnitude of this response differs considerably [9]. Given that aging microglia exhibit greater inflammatory alterations [292], it is possible that these cells carry out deleterious immune responses following ischemic injury that are not evident in young animals.

#### Pathogenic role of microglia in the context of diabetes co-morbidity

Importantly, approximately 70% of stroke patients have pre-existing conditions, such as diabetes and high blood pressure, with diabetes being associated with significantly worsened recovery and post-stroke cognitive impairment [168, 315]. To study the link between microglia, stroke, and diabetes, Jackson et al. [168] used an intracerebroventricular injection of short hairpin RNA to silence *Csfr1* and ablate microglia prior to the induction of stroke via transient MCAO in young mice with diet and streptozotocin-induced type 2 diabetes mellitus [168]. It is known that an increased ratio of pro- to anti-inflammatory microglia in diabetic animals correlates with the development of post-stroke cognitive impairment [167]. In contrast to studies in mice without comorbidities, microglial depletion in diabetic animals decreased ischemia-mediated brain injury (Table 4), indicating a diminished protective role of microglia in diabetes [168]. This study emphasizes the importance of pre-existing co-morbidities in both understanding the role of microglia in stroke pathology and the potential translation of microglia-targeted treatments for this disease. Microglia studies in older mice with diabetes or other systemic cerebrovascular illnesses, such as hypertension or atherosclerosis, are critical for translating these findings to clinical practice, as the majority of ischemic strokes occur in older individuals [24, 95] with underlying cerebrovascular disease [14].

### Monocytes in stroke

#### Monocyte contribution to tissue damage and repair

The immune response to ischemic injury critically involves the infiltration of peripheral immune cells, including monocytes and neutrophils [116, 171]. Monocyte activation has a significant impact on disease outcome following CNS injury, in particular, the secondary damage following ischemic stroke. The differential protective or pathogenic roles of monocytes in experimental models of ischemic stroke, as well as their associated phenotypes, are compared in Table 4.

In response to ischemic injury, infiltrating monocytes may adopt functional phenotypes facilitating debris clearance, wound healing, and tissue repair (i.e., pro-resolution phenotypes) [360]. However, these cells may also adopt a pro-inflammatory phenotype whose sustained production of inflammatory mediators risks secondary damage to surviving tissue. Following ischemic injury and haemorrhagic stroke, for instance, monocytes recruited to the injured brain cause inflammatory damage and functional impairment via their sustained production of TNF and IL-1β [81, 143, 192, 269]. The pathological role of these cells is further emphasized by studies in CCR2-deficient mice in which the reduced monocyte influx into the injured brain substantially reduces inflammation and secondary damage [81, 192]. However, the same cells subsequently contribute to tissue repair. In CCR2-deficient mice or following early monocyte depletion by clodronate liposomes in stroke, the resultant reduced expression of tissue repair proteins, including TGF-β1 and collagen-4, was associated with neurovascular unit instability and later haemorrhagic transformation [127], demonstrating the importance of these cells in healing after stroke.

Thus, although a pro-inflammatory monocyte phenotype is thought to contribute to secondary damage, this functional specification can also activate immune system processes involved in wound healing and tissue repair [360]. This may involve other cell types. For instance, microglial activation and proliferation is necessary in the formation of a glial scar around injured tissue [271]. Using two experimental models of ischemic stroke (photothrombosis and transient MCAO), it was recently suggested that monocytes expressing inflammatory mediators IL-1β and *Cybb*-derived reactive oxygen species (ROS) facilitated microglial proliferation and enhanced wound healing [343]. This damaging phenotype was, however tightly regulated. Following their initial pro-inflammatory activity, monocytes were physically enclosed in a glial scar facilitated by chemotactic CXCR4-CXCL12 signalling, thereby limiting monocyte-driven secondary injury [343]. Notably, the tissue infiltration patterns of monocytes and microglia differed among stroke models, potentially suggesting that multiple experimental models are necessary to fully comprehend the activities of these two cell types in disease. Nonetheless, the beneficial outcome of myeloid-mediated inflammation in these ischemic stroke models contrasts with that of experimental models of MS or viral encephalitis, where a sustained oxidative stress phenotype is detrimental [122, 224], and points towards the importance of local regulatory mechanisms in preventing aberrant inflammatory damage.

The seemingly paradoxical protective and pathogenic functions of monocytes may be further explained by the recruitment of multiple populations that differentially contribute to damage and repair as monocytes. Such populations may be differentially recruited to the site of ischemic injury in a manner similar to that occurring in mechanical injury: following mechanical spinal cord injury, ‘protective’ pro-resolution monocytes (Ly6C^lo^CX3CR1^hi^) were thought to gain access to the CNS from the choroid plexus and migrate to the injured spinal cord parenchyma through the central canal, whereas detrimental pro-inflammatory monocytes (Ly6C^hi^CCR2^+^) were proposed to transmigrate via the spinal cord leptomeninges in a CCL2-dependent manner [281]. The differential expression of Ly6C may underlie these differences, as high expression of Ly6C would better enable the emigration of inflammatory cells from the CNS leptomeningeal vasculature [310]. CNS region-specific signals may also shape their eventual functions within the CNS, with those trafficking through the choroid plexus and cerebrospinal fluid encountering stimuli that shape more anti-inflammatory roles [281]. Whether these CNS environments shape distinct cellular functions of infiltrating cells following stroke warrants further investigation. Indeed, in other disease models such as EAE, it is evident that the cytokine milieu in the choroid plexus shapes the pro- or anti-inflammatory specification of infiltrating monocytes [164].

It is crucial to remember that inflammation can be both harmful and beneficial to recovery from ischemic injury, and that any therapeutic window may be limited to a specific time period when inflammation is pathogenic. Although this therapeutic window is well-defined experimentally [171], the time course of lesion evolution is protracted in humans and complicated by recurrent ischemic episodes and systemic co-morbidities [223]. The roles, phenotypes, and transcriptomic profiles of both microglia and monocytes in animal models that better re-create the disease co-morbidities that lead to, and modulate the immune response to, ischemic stroke are therefore critically needed.

## Neurodegeneration

Alzheimer’s disease is a chronic neurodegenerative disease causing progressive and irreversible cognitive decline. Disease onset can occur at < 65 (familial early-onset) or > 65 years of age (sporadic late-onset), with the latter afflicting more than 95% of patients [87, 213]. Histopathologically, AD is characterized by the formation of intraneuronal neurofibrillary tangles consisting of the hyperphosphorylated microtubule protein, tau, and extracellular deposits of plaques of amyloid-β (Aβ), a breakdown product of the transmembrane amyloid precursor protein (APP) [241]. A transgenic system that reproduces either of these two characteristic AD pathologies is the most common approach to modelling AD in mice. In transgenic mouse models, Aβ plaques are generally generated by mice over-expressing five human familial AD gene mutations (*i.e.,* 5xFAD mice) or the APP-presenilin 1 gene (*i.e.,* APP-PS1 mice) [142]. Tauopathy is generally modelled in mice over-expressing human transgenic tau. However, these animal models do not reflect the entire biology of human AD, which presents inherent limitations to the translation of laboratory findings into a clinically relevant setting.

### Relevance of murine models to human AD

Modelling all aspects of AD pathology in an experimental animal setting is difficult. This can be attributed in part to (1) the disease being unique to humans, as far as we know, (2) the multifactorial nature of AD having both genetic and environmental etiologies, and (3) the high variability in disease onset, development and progression among patients [86].

Currently, transgenic mouse lines are the most common experimental model for AD, despite each only recapitulating one or few aspects of the human pathology. These transgenic mice express human genes that result in the formation of amyloid plaques (APP or PSEN1) and/or neurofibrillary tangles (MAPT), with other aspects of human pathology incompletely represented, such as memory-associated cognitive impairments, contribution of aging, neuronal loss, widespread neurodegeneration and regional brain atrophy [86, 325]. It is thus little wonder that the success rate of drugs that have gone through clinical trials from 2002 to 2012 show a high failure rate (~ 99.6%) [69]. In 2020, there was  ~ 40-fold and ~ threefold decrease, respectively, in the number of drugs in development for AD, compared to those in development for malignant neoplasms and diabetes [68]. The high failure rate of agents in clinical trials and relatively limited drug development for AD reflect the biological disconnect and thus restricted translatability between mouse models and human pathology. This is further exacerbated by the limited availability of human biomarkers, longer trial durations and prohibitively high trial costs [68, 86, 102]. Also feeding into this is the fact that mouse models are largely based on the expression of mutations associated with familial early-onset AD, while drugs tested in clinical trials are mainly in patients with sporadic late-onset form of AD. It follows that conclusions based on the interpretation of, and extrapolation from mouse findings should be carefully contextualized and made with caution. However, importantly, notwithstanding their limitations, mouse models have provided substantial insight into the contribution of specific features (i.e., amyloid or tau) in the pathogenesis of AD. Thus, understanding the precise limitations of each model is necessary to better translate laboratory findings into clinically relevant settings.

However, with the increasing incidence of AD and with it the increased estimated global expenditure needed for AD care, there is an urgent need to develop models that better reflect the human disease and inform effective preventive and interventive treatment [258]. Improved models of AD are underway. These include the use of primates [276], brain organoids [249] and further transgenic mouse lines. To support this, the National Institutes of Health established the Model Organism Development & Evaluation for Late-Onset Alzheimer’s Disease consortium with the aim of developing 50 new rodent models based on human datasets and that better reflect AD pathology, with all data generated being publicly available.

### Microglia in Alzheimer's disease

In 1908, Alois Alzheimer argued that microglia were implicated in the pathogenesis of AD, based on observations of altered cell morphology in CNS tissue from patients [7]. Changes in microglia/macrophage morphology and density have been further linked to a variety of neurodegenerative illnesses, including Parkinson's disease (Fig. 1c) and amyotrophic lateral sclerosis (Fig. 1d–f), in addition to AD (Fig. 1g–i). The likely involvement of microglia in AD pathology, however, was not fully appreciated until the development of genome-wide association studies. This revealed that 60.4% of AD-risk single-nucleotide polymorphisms are highly expressed by microglia [212, 364], including *Apoe, Trem2, Abca7, Cd33* and *Cr1* [221]. More recently, single nuclei RNA sequencing on AD brains showed that out of seven cell types, gene expression changes were shown only in microglia [119]. Given the demonstrable link between microglia-expressed genes and AD, many studies have attempted to elucidate their precise functions in this disease using murine models, although remarkably, genetic studies have implicated microglia in almost every defined neurodegenerative disorder [221].

#### Role of DAMPs and microglia in promoting low-grade inflammation and neurodegeneration

While DAMPs are thought to be beneficial in acute inflammation by protecting the CNS from pathogen evasion and tissue damage [178], in AD, the chronic release of DAMPs, mitochondrial-released DAMPs and pro-inflammatory molecules contributes to the onset and progression of neurodegeneration [57, 76, 97, 149, 194, 270, 304, 333, 345]. The underlying mechanism likely involves the binding of these molecules to microglia-expressed receptors, causing self-perpetuating microglial activation with sustained release of pro-inflammatory mediators. This is thought to divert microglia from performing house-keeping functions, promoting a low-grade inflammatory state and CNS damage [149, 333]. Collateral damage to proximal neurons perpetuates this cycle via the further release of DAMPs [57] and potentially the progression of neurodegeneration. Indeed, the DAMP, high-mobility-group box protein 1 is elevated in the sera of AD patients and correlates with levels of Aβ in the CNS [97]. Notably, microglial-expressed Mac-1, a potential receptor for high-mobility-group box protein 1, is increased in expression in AD brains [5] with ligand–receptor binding resulting in the production ROS and pro-inflammatory molecules [110]. This suggests a role for this DAMP in perpetuating chronic inflammatory signalling in the CNS and the progression of AD [57].

It is unclear what causes the initial release of these molecules, which sustains a low-grade inflammatory response in the brain. However, studies have shown that cellular aging, infection, traumatic brain injury, systemic inflammation, chronic inflammatory diseases, obesity and poor oral health occurring during middle adulthood can lead to increased pro-inflammatory signals in the periphery, which may contribute to glial activation and the onset and/or perpetuation of neurodegeneration [93, 105, 149, 333]. Indeed, lipopolysaccharide (a major gram negative bacterial cell wall component)-induced inflammation in mice with amyloidosis or tauopathy increased neuropathology [187, 283]. Targeting neuroinflammatory processes and the activation of innate immune responses which are paradoxically protective in acute inflammatory scenarios may provide a necessary therapeutic approach to AD.

#### Role of microglia in the formation and clearance of Aβ plaques

Both in human AD and its mouse models, microglia surround Aβ plaques [216, 226, 365]. Although more human studies are required, the local function of these cells appears to be bidirectional, with microglia having both protective and pathological functions. Microglia are thought to contribute to the formation of plaques while also compacting and clearing Aβ deposits to prevent damage to neighboring neurites. See Table 5 for a comparison of the protective and pathogenic roles of microglia in AD, as well as their disease-associated transcriptomes and immune profiles.

Microglia contribute to the phagocytic and enzymatic clearance of Aβ plaques [82] in concert with resident perivascular macrophages [311, 356]. These cells also have a role in compacting Aβ to potentially prevent their spread and impact on local neurites. Indeed, microglia depletion in four-month-old humanized APP mutant knock-in homozygote (APP^NL−G−F^) mice resulted in reduced plaque compaction and increased plaque volume, number, and size, demonstrating that microglia play an essential role in plaque compaction between four to six months of age in mice [61]. The increased Aβ burden with age suggests that microglia become senescent and are unable to phagocytose Aβ or that their ability to do so becomes latent [101, 302]. Indeed, microglia ablation in AD mouse models at 10 months of age had no impact on plaque burden, suggesting that these cells are dispensable for Aβ clearance and plaque formation in the late stage of disease [296]. To overcome this so-called latency, administration or expression of IFN-γ, TNF or IL-6 in the mouse CNS reduced Aβ deposition [48–50], potentially stimulating microglial phagocytosis and clearance of Aβ from the brain.

**Fig. 4:**
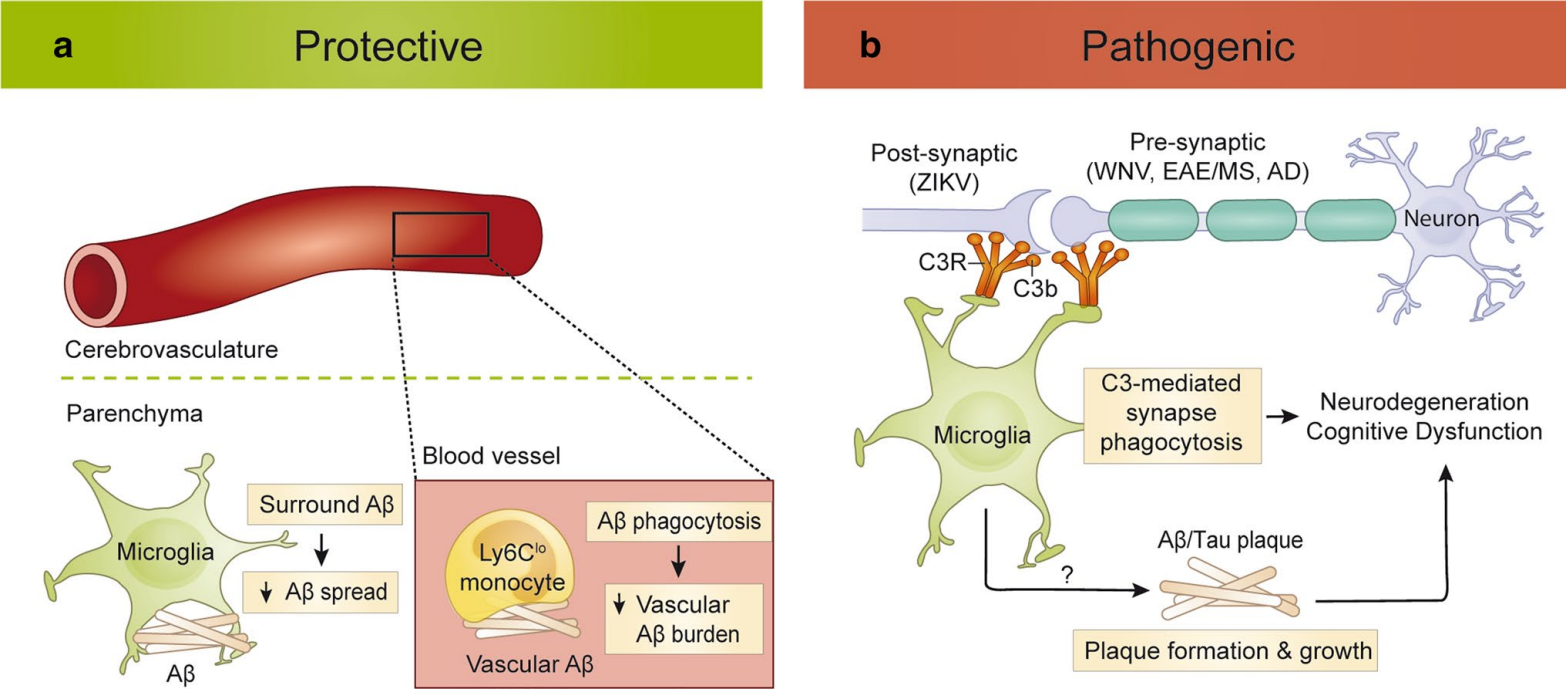
Protective and pathogenic roles of monocytes and microglia in neurodegeneration. **a** Protective functions. In AD, microglia and Ly6C^lo^ monocytes reduce parenchymal and vascular Aβ spread, respectively. **b** Pathogenic functions. Microglia phagocytose C3-tagged synaptic termini via C3R, leading to neurodegeneration and cognitive dysfunction in post-infectious encephalitis, AD, EAE and MS. *Aβ* amyloid beta; *AD* Alzheimer’s disease; *C3* complement component 3; *C3R* complement component 3 receptor; *EAE* experimental autoimmune encephalomyelitis; *MS* multiple sclerosis; *ZIKV* Zika virus

In contrast to the protective roles of microglia in AD, such as the clearance and compaction of Aβ, microglia can also contribute to Aβ deposit formation early in disease. Microglial ablation using PLX3397 in two-month-old 5xFAD mice [294] or PLX5622 in 1.5-month-old 5xFAD [295] mice prevented intraneuronal amyloid and neuritic plaque formation [294] and significantly reduced plaque formation [295], respectively (Fig. 4 and Table 5). Ablation of microglia in younger mice before development of cerebral amyloidosis thus reduces Aβ deposition [294], while ablation of microglia later in disease (*i.e.,* in 10-month-old 5xFAD mice) [296], does not alter Aβ load. Interestingly, a fraction of microglia in the thalamus were resistant to 10 weeks of PLX5622 treatment and were associated with the few plaques that had formed [295]. Thus, even a small number of microglial cells are sufficient to initiate plaque pathology. It has been suggested that microglial lysosomes provide a suitable environment for Aβ aggregation. Indeed, Aβ aggregates were found within the lysosomes of microglia in brain regions where plaques were not yet formed in non-PLX5622-treated 5xFAD mice [295]. However, Aβ deposition can also be observed in regions of the brain devoid of microglia, including the dura mater, choroid plexus and perivascular space [71, 253], suggesting that microglia may not be the only cells contributing to Aβ plaque aggregation.

#### Role of microglia in tau-related pathology

It has recently been suggested that AD progression is better correlated with tau pathology, neuronal degeneration, Aβ spreading and synapse loss, rather than Aβ detection alone [222]. While the role of microglia in tau-related pathology is relatively understudied compared to their role in Aβ formation and clearance [328], it has been suggested that microglia may also contribute to tau pathology. Microglial expression of pro-inflammatory mediators and release of extracellular vesicles have been shown to promote the pathological spread of tau.

Consistent with this, tau pathology was reduced by inhibiting microglial activation via overexpression of CX3CL1, a chemokine released by neurons that binds to microglial-expressed CX3CR1 [240] or by administration of anti-inflammatory drugs [113]. Conversely, promoting microglia activation via lipopolysaccharide- or virus-induced inflammation promoted tau pathology [187, 304]. Fascinatingly, microglial activation can even induce tau accumulation in wild-type mice with no induced tauopathy [112], strongly supporting the importance of microglia-mediated inflammation in initiating tau pathology. Although the exact mechanisms that cause microglial activation are unknown, it is possible that the low-grade chronic inflammation (described above) that occurs in middle-aged individuals promotes neuroinflammation and the progression of tau pathology [57, 97, 194, 304, 333]. Tau oligomers and monomers can also activate NLRP3 inflammasomes, further perpetuating microglial activation [162] and potential progression of tau pathology. While more human studies are required, it is clear from murine models that microglia have an indirect role in promoting tau accumulation via expression of pro-inflammatory mediators.

Microglia have also been implicated in the pathological spreading of tau via the release of extracellular vesicles [12, 61, 328]. While human and microglia can internalise aggregated or hyperphosphorylated tau for protein degradation, they can also release incompletely degraded or un-neutralised forms back it into the extracellular space, promoting the spread of tau [12, 32, 36, 108, 153]. Indeed, depletion of microglia in mice injected with tau-expressing adeno-associated virus reduced tau propagation, which was hypothesized to occur by ablating microglial-mediated phagocytosis and subsequent re-secretion of tau-laden exosomes for the pathological spread and accumulation of tau.

To study the mechanistic link between microglial-mediated tau propagation and amyloid accumulation, microglia were depleted in APP^NL−G−F^ mice injected with tau-expressing adeno-associated virus [61]. Intriguingly, microglia were shown to be three times as phagocytic and three times more likely to release tau-containing extracellular vesicles when situated near amyloid plaques [61]. This was associated with enhanced tau propagation in mice with amyloidosis, compared to wild-type mice without amyloidosis [61], highlighting the importance of studying both amyloid- and tau-related pathological features of AD in one model. Interestingly, microglia depletion had no effect on tau in aged mice [27]. This could suggest that microglia become senescent and cannot phagocytose tau, potentially leading to decreased extracellular vesicle production and tau spread. However, results from this study may have been confounded by incomplete microglial depletion, which was only ~ 30% [27].

Tau accumulation is frequently associated with synaptic loss. Microglia can phagocytose tau-laden synapses or entire neurons [36, 75], promoting neurodegeneration and memory loss. As previously mentioned, microglial-expressed complement components, including C1q, are thought to bind to synapses and enable phagocytosis by microglia [75, 103]. While more investigation in the human is required to confirm microglial association with C1q-mediated tagging of synapses, in normal aging [300] and in tauopathy patients [75], C1q is dramatically upregulated and is shown to co-localize with hyperphosphorylated tau, plaques and neurofibrillary tangles in AD brain sections [1, 219, 282]. Together, this work suggests that while microglia attempt to perform physiological functions, such as clearing pathological tau and Aβ aggregates, these proteins may be incompletely degraded and re-secreted into the extracellular space to spread protein accumulation. These proteins can also bind microglial receptors, promoting pro-inflammatory signalling and diverting microglia from house-keeping functions, thereby causing a self-perpetuating chronic inflammatory response that induces further protein accumulation and tau seeding.

#### The ‘universal’ neurodegenerative microglial phenotype

Recent transcriptomic studies have identified a subset of microglia that adopt unique transcriptional and functional signatures, termed ‘disease-associated microglia’ (DAM) [179] or ‘microglial neurodegenerative’ (MGnD) phenotype during neurodegeneration [191]. These microglial signatures are characterised by the downregulation of homeostatic genes (*P2ry12, Tmem119, Sall1* and *Cx3cr1*) and upregulation of an inflammatory program (*Trem2, Apoe, Axl, Lpl* and *Clec7a*) (Table 5), several of which have been identified in genome-wide association studies linked to human AD. The DAM phenotype has been proposed to represent a universal and intrinsic microglial response program to CNS disease, since it appears to be retained across a number of neurodegenerative and non-neurodegenerative diseases [74, 291]. However, it remains unclear if this intrinsic response program specifically identifies a functionally meaningful subset, and whether it is beneficial or detrimental to disease pathogenesis.

Several studies have suggested that the microglial DAM signature is protective in neurodegeneration. This has been highlighted by the importance of TREM2, a receptor expressed by DAM/MGnD, which exerts its protective function in AD via ligation of ApoE (apolipoprotein E), which binds to loose Aβ aggregates, or direct ligation of Aβ oligomers [286, 295, 338, 357, 366]. This process enables lysosomal degradation and phagocytotic trimming of Aβ by surrounding microglia, which may be crucial to the compaction (but not removal) of developing Aβ deposits to protect neurons from injury (Table 5). Accordingly, AD patients carrying mutations in TREM2 genes display less compact Aβ plaques and fewer plaque-associated microglia but enhanced tau pathology and neuritic plaques [191, 361]. Similarly, in mice with cerebral amyloidosis, genetic ablation of TREM2 abrogated the MGnD phenotype and reduced the microglial response to plaques [191]. This supports a protective role for the DAM/MGnD microglial phenotype, with TREM2-dependent functions potentially required initially to prevent seeding of plaques, whilst later enhancing the consolidation of Aβ into compact aggregates to protect neurons from Aβ-induced injury. This protective function appears to be independent of other microglia-mediated functions which are detrimental to neurons, such as their contribution to synapse loss.

Other studies instead argue that the microglial DAM/MGnD phenotype is damaging. It is known that homeostatic microglia support neuron maintenance [211, 306]. Thus, the phenotypic transition observed in DAMs may harm neurons by causing low-grade inflammation and lowering neurotropic support, which may contribute to increased tau phosphorylation [82]. In support of this, gene expression analysis showed that pathways related to neuronal function (i.e., glutamate receptors, synaptic vesicles, and neuronal membranes), were downregulated in the hippocampus of 5xFAD mice [295]. This pattern was reversed in the absence of microglia, suggesting that these cells restrict gene expression pathways crucial to neuronal function in AD [295]. Furthermore, complement activation, which is important for microglial phagocytosis, can exacerbate synaptic loss [152, 284, 291] and contribute to memory loss and cognitive decline [75] (Table 5). Consequently, microglia depletion before or after the formation of Aβ pathology in mouse models of AD resulted in improved contextual memory, further implicating microglia in neuronal and/or synaptic loss [294–296].

The ability of microglia to return to homeostasis during AD may be impaired by the downregulation of the microglial receptors CX3CR1 and CD200R, which bind to neuronal ligands and act as immune checkpoints that maintain microglia homeostasis [30]. Furthermore, DAM-expressed factors, such as apolipoprotein, colony-stimulating factor 1 (CSF1) and secreted phosphoprotein 1, act as autocrine ligands on DAM-expressed receptors, together sustaining and perpetuating the neurodegenerative phenotype [291]. Thus, both protective and pathogenic functions are likely to be carried out simultaneously by the DAM phenotype.

#### Relevance of DAM/MGnD to human AD

The importance of the DAM/MGnD phenotype must be more carefully considered in the context of human pathology. In contrast to mouse studies, single cell nuclei extraction from frozen human brain tissue revealed 13 distinct microglial clusters [119]. While a population of clusters, namely AD1, closely reassembled the mouse DAM/MGnD phenotype, it was only abundant in human samples containing Aβ and not tau. Conversely, AD2 did not overlap with AD1 and was more frequently observed in human brain samples showing both tau and amyloid pathology [119]. These data highlight the usefulness of amyloidosis mouse models in exploring the human AD1 (DAM-like) phenotype; however, it demonstrates that the additional presence of tau in the CNS could induce a different microglial phenotype. This may explain why single-cell RNA-seq analysis on 16,242 live microglial cells isolated from the aging and AD human brain demonstrated an overall weak correlation with the DAM signature [246]. Together this work demonstrates, firstly, that there exist other microglial subsets in AD besides the DAM/MGnD phenotype, and secondly, that human microglia are heterogenous, thus limiting the translation of the DAM signature from animal models. This therefore highlights the need for improved mouse models that better replicate human AD and/or that further investigation is required of other non-DAM/MGnD phenotypes in the diseased mouse brain.

In support of these conclusions, other microglial phenotypes beyond the DAM/MGnD have been discovered in murine models of CNS pathology [106, 264]. Perhaps these different microglial transcriptional programs feature in different aspects of AD pathology, as shown in a human study where it was thought that one microglial transcriptional program contributed to tau pathology and two others to β-amyloid pathology [252].

The role of microglia in AD is clearly complex. Nevertheless, despite the disconnect between human and mouse models, without experimental modelling, we would never have understood certain aspects of the human pathology. Current murine models suggest that microglia contribute to the formation of plaques and tau aggregates, as well as neuronal damage and synapse loss. On the other hand, microglia have a role in phagocytosing pathological tau and compacting Aβ aggregates to prevent local neuritic damage. This may reflect an overall attempt to protect the brain by slowing down damage, but ultimately persistent and low-grade inflammatory signals, as well as the continued accumulation of Aβ and tau aggregates, interfere with CNS functions. Taken together, several lines of evidence indicate that microglia play a substantially pathogenic role in AD, which appears to be in stark contrast to the overall effect of these cells in viral encephalitis and stroke. One obvious explanation for this lies in the acute versus chronic nature of these diseases.

### Monocytes in Alzheimer's disease

The role of peripherally derived monocytes in neurodegenerative disorders such as AD has long been debated. It is unclear if and to what extent monocytes infiltrate the brain in neurodegenerative disorders, which feature low-grade inflammation rather than the severe, acute inflammation seen in viral encephalitis and ischemic stroke. This problem is complicated further by the difficulties of distinguishing peripherally-derived monocytes from resident microglia in human tissues histologically (Fig. 1g–i), as previously noted in other diseases.

#### The contentious involvement of monocytes in AD pathology

Findings supporting a neuroprotective role for monocytes in AD pathology are conflicting and have been confounded by the use of experimental systems that condition the brain for monocyte infiltration, thereby preventing the accurate representation of disease pathophysiology. For instance, studies utilizing whole body irradiation chimeras, an experimental technique that is now recognized to condition the brain for monocyte infiltration [2, 3, 230], as well as transplant of BM-derived cells, reported that Ly6C^hi^ monocytes infiltrate the AD mouse brain and accumulate around Aβ plaques. These infiltrating cells were thought to ameliorate disease evolution by phagocytosis and clearance of Aβ from the brain parenchyma [287] or by promotion of CCR2-mediated microglial accumulation in amyloid plaques and degradation of Aβ [89]. Although it is unclear whether transplanted cells or monocytes engrafted the AD brain, this hypothesis was supported by observations that CCR2 deficiency worsened AD pathology [89, 238], presumably due to an inability to recruit Ly6C^hi^ monocytes from the BM [280]. However, subsequent parabiosis experiments using head-protected irradiation chimeras [229] or chemotherapy-induced myeloablation [227], which avoid the brain conditioning discussed above, demonstrated that Ly6C^hi^ monocytes are not recruited to the brain parenchyma during the course of AD. Subsequent studies further revealed that parenchymal Aβ burden is unaltered in CCR2-deficient mice [229, 238], further exonerating Ly6C^hi^ monocytes from the responsibility for clearing Aβ in AD. Moreover, in a transgenic *CD11b-HSVTK* mouse model, in which ganciclovir administration “paralyses” CD11b^+^ cells including microglia (i.e., where proliferation and activation is blocked), engrafted BM-derived monocytes failed to accumulate around Aβ plaques and did not reduce Aβ plaque burden [262]. Taken together, these findings suggest that Ly6C^hi^ monocytes either do not infiltrate the brain parenchyma in large enough numbers to alter parenchymal Aβ burden, or these cells do not participate in Aβ phagocytosis or clearance once in the brain.

Despite their lack of local involvement in the brain, blood monocytes may play an important role in the periphery. It is estimated that approximately 50–62% of Aβ diffuses into the blood [252, 261] and is thus cleared from the CNS [336, 353]. Monocytes in the peripheral circulation showed a reduced Aβ uptake capacity with age and in AD patients [56], implying that impaired Aβ uptake by monocytes may play a role in the aetiology of AD. Several human AD risk gene variants have been linked to monocytes, including those related with *TREM2* and *CD33* [34, 52], the latter of which is linked to diminished Aβ internalization by peripheral monocytes [34].

The importance of peripheral monocytes in Aβ clearance is supported by experimental studies demonstrating that peripheral Ly6C^lo^ monocytes likely contribute to Aβ clearance from the cerebral vasculature, thereby indirectly reducing parenchymal Aβ burden [227] (Fig. 4). By intravital two-photon imaging, patrolling Ly6C^lo^ monocytes were shown to participate in Aβ clearance from the leptomeningeal vasculature [227]. These cells selectively crawl along Aβ-laden veins and scavenge Aβ from the lumen. Depletion of these cells by deletion of *Nr4a1*, a transcription factor controlling the differentiation and survival of Ly6C^lo^ monocytes [144], corresponded with increased Aβ deposits in the cortex and hippocampus [227], implicating a critical role for vascular Aβ clearance by patrolling monocytes. The protective function of Ly6C^lo^ monocytes in AD may also explain why CCR2 deficiency exacerbates disease severity [89, 238], as Ly6C^lo^ monocytes, which differentiate from recruited CCR2^+^Ly6C^hi^ monocytes in the bloodstream, would no longer be recruited to this function [323, 359]. On the other hand, yolk sac-derived perivascular macrophages also clear Aβ from the vasculature [147] in a CCR2-dependent manner [229], and impaired Aβ clearance by these cells would presumably also contribute to the accelerated disease progression observed in CCR2-deficient mice.

In addition to their role in Aβ pathology, monocytes may participate in the low-grade, systemic inflammation that can accompany aging and cognitive decline in AD. Although overall peripheral blood monocyte counts do not appear to vary over disease course, there are population shifts in monocyte subsets in patients with AD [312]. This is evidenced by a decrease in classical monocytes (Ly6C^hi^ in mice) and an increase in intermediate and non-classical monocyte populations (Ly6C^lo^ in mice) with disease progression, which coincided with a shift in monocyte pro-inflammatory gene expression (including *IL6*, *TNF* and *ILLB*) [312]. Moreover, IL-33 signalling shown to reduce synaptic impairment and subsequent memory deficits in APP/PS1 mice was impaired in lipopolysaccharide-primed and Aβ-stimulated monocytes from patients with AD dementia [275], further suggesting that an altered monocyte phenotype may contribute to AD pathology.

## Demyelinating disease

Multiple sclerosis is an inflammatory demyelinating disease of the CNS, thought to be of autoimmune aetiology, characterised by chronic neuroinflammation and neurodegeneration. It involves the progressive loss of oligodendrocytes and neurons, resulting in motor and cognitive deficits [322, 326]. Clinical displays of MS are highly heterogeneous and can comprise clinically isolated syndromes, primary progressive forms and relapsing–remitting patterns, the latter causing ~ 85% of initial cases and potentially developing into secondary progressive MS over a span of 10–15 years [309, 346]. Relapsing–remitting MS is characterised by episodes of neurological impairment followed by stages of remission and partial neurological recovery, while progressive MS forms involve progressive neurological impairment without phases of remission [326, 346]. The extensive infiltration and activation of peripheral and resident immune cells in MS suggests these cells are key mediators of lesion formation [77, 210, 278] (Fig. 1j-l). However, mirroring the heterogeneity of clinical presentation, different classes of lesion exist, depending on disease type, duration, cellular players and anatomical compartment [205, 256], making this complex scenario challenging to mimic in a single animal system [40, 186, 196].

### Relevance of murine models to human MS

Similar to other CNS pathologies, MS is a uniquely human disease, and as such can only be partially modelled in experimental animal systems [186]. Significant caution is thus necessary in interpreting results from animal models, in particular from the widely used murine Experimental Autoimmune Encephalomyelitis (EAE) model. Inflammation in EAE is primarily mediated by MHC class II-restricted autoreactive T cells that are induced by injection of encephalitogenic antigens (e.g., myelin basic protein, myelin oligodendrocyte glycoprotein, MOG, or proteolipid protein, PLP), in conjunction with an adjuvant, or passively, via peripheral administration of encephalitogenic CD4^+^ T cells [64]. APCs play an important role in the activation of peripherally-primed T cells, resulting in an inflammatory cascade that leads to oligodendrocyte death, chronic demyelination, and neuronal loss [64, 77]. While commonly referred to as a single experimental paradigm, EAE development and pathological presentations are critically dependent on the induction method, animal strain and genetic manipulations [40, 186]. Most groups however, utilize C57Bl/6 mice, a strain typically developing an acute, monophasic disease useful to study the main inflammatory aspects of acute MS [232]. Depending on the specific model, EAE fails to fully recapitulate the complex pathological presentation of MS, including the differential distribution of lesions between brain and spinal cord, the driving lymphoid cellular components, and the relative extent of demyelination in white matter tracts [265].

Furthermore, most EAE models do not recapitulate the extensive grey matter pathology observed in patients, which show a demyelination pattern often characterized by chronic microglia activation, absence of leukocyte infiltration [46] and pronounced meningeal inflammation [156]. While novel models have recently been developed to study this type of lesion [318], other experimental approaches are better suited to model a subtype of inflammatory grey matter pathology observed in early MS cases [256] and displaying tissue-invading leukocytes supposedly driving local damage [169, 180, 203, 225]. At the same time, even though attempts to represent a chronic disease course have been made [23], and mouse strains, such as Biozzi ABH [6] and A.SW [314], show a clinical development resembling progressive MS, most EAE models are not suited to mimic primary/secondary progressive pathology or general disease chronicity. For instance, EAE induction in non-obese diabetic (NOD) mice, often claimed to represent the clinical course of progressive MS, can be used to study some neurodegenerative aspects of the disease, but overall it lacks evidence as a valid chronic model [15]. Taken together, the need remains for novel experimental approaches that better model progressive and chronic MS.

Notwithstanding these drawbacks, EAE has proved of unmatched value for the understanding of several pathological mechanisms evidently occurring in the human disease; furthermore, it still represents the best platform to test novel therapeutic approaches [186]. Research in EAE models has for instance directly led to the development of the disease modifying therapies, glatimer acetate and natalizumab, while other drugs, such as fingolimod, dimethylfumarate and alemtuzumab, have been tested and mechanistically explained, thanks to this model [186]. On the other hand, an important number of therapies stemming from EAE studies, in particular most approaches involving manipulation of cytokine actions, have surprisingly and consistently failed successful clinical application, once again showing the context-dependent limitations of EAE [265].

Other aspects of MS, such as demyelination in the relative absence of adaptive immunity-driven inflammation, are alternatively studied using chemical- (i.e., lysolecithin-induced) [352] or toxin- (i.e., cuprizone) mediated demyelination models [257]. Other groups have instead used diphtheria toxin-induced death of oligodendrocytes to mimic the formation of lesions in some early MS cases, showing oligodendrocyte apoptosis in the absence of immune cell infiltration [17, 202, 313]. The spontaneous development of demyelination seen with viral infection, e.g., TMEV infection, is also a useful model to study specific neurodegenerative characteristics seen in MS [118].

### Microglia in multiple sclerosis

Akin to many neuroinflammatory diseases, the “empirical observation of microgliosis” implicates a role for microglia in MS pathogenesis [132]. Indeed, the number of ‘activated’ microglia and macrophages correlates with axonal damage in MS lesions [31, 99, 267, 327]. Interestingly, however, gene expression patterns from human brain data at the tissue-level did not show an enrichment in MS susceptibility genes [161]. Extending the analysis to include data generated from human induced pluripotent stem cell-derived neurons, astrocytes and microglia, revealed the enrichment of MS susceptibility genes in microglia but not astrocytes or neurons [161]. The *CLECL1* locus implicated a role for microglia in MS pathogenesis, which was underestimated in tissue-level brain profiles. The role of microglia in MS however remains unclear and might be dependent on the type and chronicity of distinct lesions. Several insights into microglia function could be inferred from animal studies, despite obvious limitations and potential species-related cellular differences [138].

#### Duality of microglial roles in immune or chemical demyelination

**Fig. 5:**
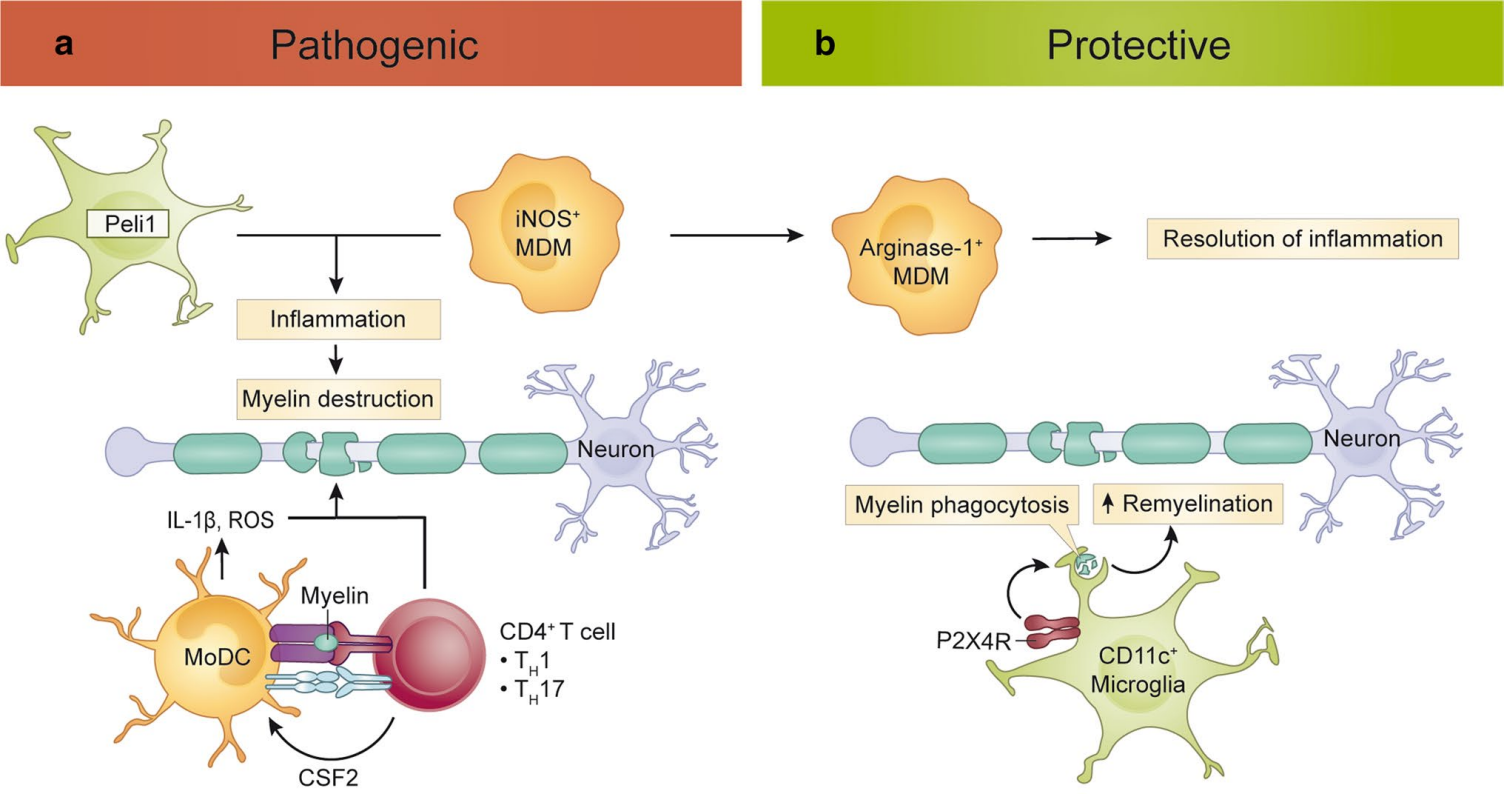
Protective and pathogenic roles of monocytes and microglia in autoimmune neuroinflammation. **a** Pathogenic functions. Peli-mediated microglial expression of pro-inflammatory cytokine and chemokines can exacerbate inflammatory damage to myelin. GM-CSF-stimulated moDC activate myelin-reactive T cells and exacerbate myelin damage via the production of ROS and IL-1β. iNOS^+^ MDM producing ROS and NO contribute to myelin destruction but may also transition to arginase-1^+^ MDM and facilitate the resolution of inflammation (**b**). **b** Protective functions. Microglia may stimulate re-myelination via P2X4R-mediated phagocytosis of myelin debris. *GM-CSF* granulocyte–macrophage stimulating factor; *IL* interleukin; *iNOS* inducible nitric oxide synthase; *MDM* monocyte-derived macrophage; *MHC* major histocompatibility complex; *moDC* monocyte-derived dendritic cell; *NO* nitric oxide; *P2X4R* P2X purinoceptor 4; *Peli* Pellino E3 Ubiquitin Protein Ligase; *ROS* reactive oxygen species; *TH* T helper cell

A detrimental role for CNS-resident macrophages in EAE was first suggested in a transgenic mouse model of so-called “microglial paralysis” (i.e., where proliferation and ‘activation’ is blocked) in lethally irradiated, *CD11b-HSVTK* mice engrafted with wild-type BM, that showed reduced EAE disease severity [150]. However, the interpretation of these data was hampered by the observation that *CD11b-HSVTK* animals also displayed reduced CNS infiltration of peripheral leukocytes. Considering that CCR2 blockade/deficiency [2, 228], clodronate liposome or immune-modifying nanoparticle administration, all of which abrogate CNS infiltration of Ly6C^hi^ monocytes and result in attenuated EAE signs [121, 233], this implies a key function of Ly6C^hi^ monocyte-derived cells in EAE development, leaving a potential pathogenic or protective role for microglia in the *CD11b-HSVTK* model unresolved. Subsequently, a study in which mice were fed high- and low-dose PLX5622 post-EAE induction showed improved disease signs without affecting peripheral immune cell populations. PLX5622-treated mice also showed an increase in mature oligodendrocytes, suggesting a role for microglia in regulating oligodendrocyte maturation [243] (Fig. 5 and Table 6).

The exact role of microglia in EAE is unclear. However, it appears that microglial ‘activation’ and production of pro-inflammatory mediators have a role in the pathogenesis of the disease (Fig. 5). Pellino-1-deficient (*Peli1*^*−/−*^) mice were refractory to EAE induction [354] (Table 6). Peli1, expressed by microglia in the brain and upregulated during EAE, is an E3 ubiquitin ligase that enhances Toll-like receptor/MyD88 signaling via the degradation of the adaptor protein TRAF3 and the activation of the mitogen-activated protein kinase, with the consequent induction of several pro-inflammatory cytokine (*Il1b, Tnf, Il12b*) and chemokine (*Cxcl1, Ccl2, Ccl20*) mediators. However, other CNS-resident and infiltrating macrophage populations may also express Peli1, thus deficiency of Peli1, which reduces EAE severity, implicates both microglial and non-microglial cells in the production of inflammatory mediators and the pathogenesis of EAE. Further, non-physiological effects, created in the generation of the Peli1 knockout chimera via lethal irradiation and by disrupting the function of Peli1 during development and adulthood in all cell types cannot be ruled out.

Using more selective tools to target microglia during neuroinflammation, Goldmann et al. [129] generated *Cx3cr1*^*CreER*^*:Tak1*^*fl/fl*^ mice, whereby TGF-β-activated kinase 1 (TAK1) was ablated from long-lived CX3CR1^+^ cells (which include microglia) [129]. TAK1 is a mitogen-activated protein kinase kinase kinase, upstream from nuclear factor-κβ, which upon activation can induce the production of cytokines, chemokines and adhesion molecules, contributing to inflammation and the recruitment of immune cells. Genetic ablation of TAK1 in long-lived CX3CR1^+^ cells attenuated EAE pathology [129]. This was associated with reduced cellular infiltration into the CNS, reduced myelin destruction and axonal loss, and suppression of proinflammatory genes. Using a more targeted approach to study microglia, this study supports the pathogenic role of these cells in EAE via the production of inflammatory mediators. This is in line with studies using less selective tools, i.e., the Peli1 knockout chimera, mentioned above.

More recently, using single-cell technology, a subpopulation of microglia was identified in the CNS of mice at the onset of EAE which produced ROS [224]. The production of ROS contributes to oxidative stress and injury associated with myelin damage and disease progression in MS. Using Tox-RNA sequencing to transcriptionally profile ROS-producing cells, Mendiola et al. [224] identified an oxidative stress signature (*Cybb*, *Ncf2*, *Ncf4* and *Gpx1)* shared by all MC populations and one microglia population in EAE [224]. Targeting these populations with acivicin, which targets γ-glutamyl transferase to degrade anti-oxidant glutathione, decreased disease severity in four models of EAE and a model of microglia-driven neurodegeneration (induced by a lipopolysaccharides injection in the substantia nigra) [224]. Thus, this study shows that at least one subpopulation of microglia in EAE is neurotoxic, contributing to oxidative stress and pathology.

On the other hand, using *Csf1*^−/−^ mice to reduce the number of white matter microglia, lysolecithin-mediated demyelination resulted in extensive demyelination and neuronal damage compared to WT controls, suggesting a protective role for microglia [352]. *Csf1*^−/−^ animals also showed enhanced pro-inflammatory astrocytic responses, indicating that microglia can prevent dysfunctional astrocyte responses necessary for the inflammatory-to-regenerative switch required for the initiation of remyelination (Table 6). Thus, while differences could be due to the distinct microglial depletion and/or MS models used, this study nevertheless suggests a protective role for microglia in promoting remyelination to prevent axonal damage, in contrast to the previously discussed reports.

#### Role of microglial CSF1R, TREM2 and P2X4R in EAE remyelination

As mentioned above, using high- and low-dose administration of the CSF1R inhibitor, PLX5622, Nissen et al. [243] revealed a pathogenic role for microglia in EAE, as clinical presentation of the disease was reduced upon microglial depletion. Seemingly paradoxically, stimulation of CSF1R was also protective in EAE [347]. Recombinant IL-34 or recombinant CSF1 injected intrathecally via the cisterna magna ameliorated EAE, suppressing disease progression and severity and reducing demyelination and oligodendrocyte loss (Table 6). Notably, this corresponded with the expansion of CD11c^+^ microglia, a population absent from the homeostatic CNS parenchyma but described to emerge in postnatal development in the neonatal brain, in cuprizone-induced demyelination, and in mouse models for neuromyelitis optica and AD [42, 268, 348, 350]. A CD11c^+^ microglial population further characterized as CD317^+^ MHC-II^+^ CD39^hi^ CD86^+^ was also previously identified by Ajami et al. [4] in the active phase of EAE and downregulated in the chronic phase or upon clinical recovery [4]. These cells seem to be important sources of insulin-like growth factor 1 (IGF-1) and IFN-β, thus supporting a neuroprotective role for this population [348–350]. Accordingly, deletion of IGF-1 in these CD11c^+^ cells resulted in impaired primary myelination in the developing brain [349]. Thus, reduced demyelination seen with CSF1R stimulation was suggested to be a result of the expansion of a CD11c^+^ microglia population, promoting myelination of axons.

Interestingly, EAE severity was enhanced in mice where CD11c^+^ cells were depleted using several genetic approaches [358]. This was thought to be a consequence of a reduction in inducible Tregs due to depleted CD11c^+^ DCs. However, the worsened disease outcome in the absence of CD11c may have been due to the inhibition of protective functions orchestrated by CD11c^+^ microglia or even MCs.

The purinergic P2 receptor P2X4R, highly expressed by microglia at the peak and recovery phase of disease, has also been implicated in inducing efficient remyelination [362] (Fig. 5 and Table 6). In this work, daily administration of a P2X4R antagonist from the onset of disease exacerbated clinical signs of EAE by polarising microglia into a pro-inflammatory state. Conversely, potentiation of P2X4R using ivermectin, which modulates ion conduction and channel gating of P2X4Rs, ameliorated EAE motor deficits and improved corticospinal tract function. P2X4R signalling correlated with a higher phagocytic capacity by promoting an acidic shift in lysosomes, which could favour myelin clearance required for effective remyelination [362]. This study highlights the disease-ameliorating importance of myelin phagocytosis by microglia. Similarly, microglia pre-stimulated with IL-4 ex vivo and transplanted into the CSF, also resulted in oligodendrogenesis and amelioration of EAE [43]. However, the interpretation of this experiment warrants caution, considering the non-physiological conditions associated with an intraventricular injection of in vitro-derived microglia.

TREM-2 is an important receptor involved in phagocytosis, and upon genetic absence or blockade of TREM-2, microglia fail to upregulate genes required for lipid metabolism and phagocytosis, resulting in the exacerbation of EAE [254, 255] (Table 6). These studies further support a role for microglia in the phagocytosis of myelin debris and potentially in subsequent axonal remyelination. Furthermore, gene expression studies in models of EAE support a role for microglia in apoptotic cell [199] and debris clearance [355].

The studies above demonstrate the pathogenic role of microglia in the acute phase of EAE, with polarisation of microglia to an anti-inflammatory phenotype, or expansion of a CD11c^+^ microglia population, reducing disease severity via indirectly promoting remyelination of axons and/or the phagocytosis of myelin debris. In contrast, in the toxin-induced model of demyelination in MS, microglia are protective, since microglial depletion results in an overexuberant astrocytic response and extensive demyelination.

#### Role of microglia in T cell priming and EAE pathogenesis

Microglia have also been implicated in presenting antigen to T cells in the early stages of disease, given that microglia are the first cells to take up myelin antigen [293], and can extend MHC-II^+^ cellular processes at the level of the glia limitans to potentially contact invading cells [173, 197]. T cell stimulation requires co-stimulation by CD80, CD86 and CD40, which are upregulated by microglia, although to a lesser extent than on infiltrating MCs [37]. Recently however, a series of studies utilizing different transgenic approaches elegantly showed that MHC-II expression on CNS-resident microglia/macrophages is dispensable for T cell priming and EAE pathogenesis [125, 174, 236, 351]. Instead, although this topic remains debated, the aforementioned studies implicated cDC2 type of APC in the initiation of EAE.

#### Protective role of microglia in models of progressive MS

To model the clinical presentation observed in secondary progressive MS patients, the NOD mouse immunized with MOG_35-55_ peptide has often been used. NOD mice induced with EAE typically develop secondary progression occurring ~ 30 days post immunization, following an acute attack at 14 days and remission by 20 days [309]. In this model, initiating PLX3397 treatment at 20 days post EAE induction increased mortality and exacerbated disease in immunized mice without affecting the timing of the relapse phase. PLX3397 microglia-depleted, MOG_35-55_-immunized NOD mice displayed enhanced inflammation, demyelination, axonal degeneration and a significant increase in the number of CD4^+^ and Ki67^+^ proliferating CD4^+^ T cells. These observations potentially suggest an overall protective role for microglia in the development of secondary progressive MS, in contrast to the discussed pathogenic role of microglia in the acute phase of EAE [309] (Table 6).

However, MOG-immunized NOD mice mirror only some of the biological processes observed in chronic MS patients, and caution is obviously needed when translating these results to MS [15]. In a recent study, EAE was passively induced via injection of myelin basic protein (MBP)-specific T cell blasts in LEWzizi rats. These are Lewis rats on a zitter rat background that partly mimic human aging and MS pathology, showing neurodegeneration, hypomyelination, microglia activation and iron accumulation [346]. Interestingly, there was no exacerbation of disease, compared to Lewis controls, contradicting expectations that pre-existing microglia activation and pathology (or ‘aging’) would be amplified and convert EAE into a chronic progressive disease. Pathology was instead redirected to the mesencephalon, where LEWzizi animals showed T cell infiltration and enhanced demyelination, presumably due to T cells targeting pre-damaged areas. While this study suggested that EAE pathogenesis is unaffected by microglia pre-activation and ‘aging’, further investigations and different models better suited to investigate chronic cell activation are required to confirm this.

### Monocytes in autoimmune inflammation

Monocytes are recruited to plaques in MS [193] (Fig. 1j-l) and have long been considered to play a pathological role in EAE. These cells leave the BM and migrate to the CNS prior to disease onset, with their infiltration preceding the development of paralysis and subsequent clinical manifestations of EAE [183, 228]. As the infiltration of monocytes into the CNS is mostly CCR2-dependent, CCR2-deficient mice have frequently been used to prevent their immigration into the brain and investigate their role in disease [260]. Accordingly, initial experiments with CCR2-deficient mice showed that these animals were resistant to EAE induction [98, 137, 165, 230], which suggested peripheral monocytes play a crucial role in disease pathogenesis. How monocyte-derived effector cells contribute to disease pathogenesis is less clear; such cells may contribute to pathology directly, through inflammation-mediated myelin destruction, or indirectly through activation of autoreactive T cells via antigen presentation. The protective and pathogenic functions of monocytes in MS, along with their respective immune profiles, are compared to those of microglia in Table 6.

#### An unlikely role: monocyte antigen presentation for the initiation of EAE

In EAE, the activation of encephalitogenic T cells via the presentation of the encephalitogenic antigen is a critical step in disease induction [64]. Antigen is presented by CNS-resident or immigrant APCs, which typically express MHC-II and co-stimulatory molecules, CD80 and CD86, required for CD4^+^ T helper (TH) cell activation. Antigen presentation in EAE has been attributed to DC, however, distinguishing brain-resident cDC from peripherally-derived monocyte-derived DC has challenged the accurate characterization of the antigen-presenting functions of these cell types in EAE [4, 18, 136, 174, 236]. As both peripherally-derived and CNS-resident myeloid cells have been implicated in antigen presentation to autoreactive T cells, definitive evidence of a non-redundant role for monocyte-derived APC in activating autoreactive T cells is lacking. It was recently demonstrated that, in contrast to the resident population, CNS-infiltrating myeloid cells showed prolonged interactions with T cells in the CNS and that their expression of MHC-II was essential for EAE development [174, 236]. Furthermore, myeloid-derived DC are critical for the activation and differentiation of TH17 cells [189, 231]. This was further characterized by mass cytometry demonstrating that monocyte-derived subpopulations characteristically expressed phenotypes involved in activating TH1 and TH17 cells at disease onset [4], suggesting these cells play a key role in antigen presentation.

However, the critical antigen-presenting role of moDC in EAE pathogenesis has been challenged by in vitro studies demonstrating that moDC are less capable of presenting myelin compared to cDC, and are therefore redundant in activating autoreactive T cells [125, 236]. The suggested relative inability of monocytes to present myelin peptide to autoreactive T cells has been explained by a lower expression of H2M [125], a non-classical MHC molecule facilitating the processing and presentation of myelin antigen [289]. Nonetheless, the distinction between moDC and cDC in vivo remains challenging because of overlapping phenotypic markers, complicating the resolution of their antigen-presenting functions in vivo and warranting further investigation.

#### Monocyte subsets participate in inflammatory myelin damage

In addition to possible antigen-specific T cell-mediated inflammation, non-specific inflammation also contributes to myelin destruction in MS [77]. Both CNS-infiltrating monocytes and resident microglia have been implicated in this process [133, 242], however, dissecting their respective functions in disease has proved difficult. To distinguish these cells during neuroinflammation, *Ccr2*^rfp^::*Cx3cr1*^gfp^ reporter mice were used to distinguish CCR2^+^ monocyte-derived cells from the resident CX3CR1^+^ population in EAE, based on their differential expression of RFP and GFP [355]. Using this method, resident microglia appeared to be more involved in phagocytosing myelin debris and tissue repair, whereas MDMs in the immediate vicinity of axons at the nodes of Ranvier expressed a pro-inflammatory gene signature [355], suggesting that these cells actively participate in inflammatory myelin damage.

High-parameter technologies have further elucidated the heterogeneity of monocyte phenotypes driving inflammatory damage in EAE. It was recently demonstrated that CNS-infiltrating monocytes adopt a toxic phenotype that likely mediates tissue damage [224]. Oxidative stress results from a metabolic switch during inflammatory activation that sustains the production of inflammatory mediators, such as ROS and NO [109], which is required for pathogen defence functions. In EAE, however, this oxidative stress signature is associated with inflammatory damage, a phenotype expressed by more than 50% of CNS-infiltrating monocytes [224]. The pathogenic function of this oxidative stress phenotype was emphasized by experiments demonstrating that inhibiting ROS production via inhibition of γ-glutamyl transferase, an upstream modulator of the oxidative stress pathway, reduced disease severity, decreased demyelination and axon damage, and lowered oxidative stress markers in multiple rodent models of MS [224], and may be a potential therapeutic target for human disease. However, these same cells primed to produce reactive species can be polarized from a pro-inflammatory (iNOS^+^) to a tissue-repair (arginase-1^+^) phenotype by the tissue microenvironment and move inflammatory lesions towards resolution, as demonstrated by single-cell fate-mapping. Therefore, therapeutic targeting of these cell types should be approached with caution, especially if the same monocytes expressing a pathogenic phenotype are central to the resolution of inflammation at a later stage in disease [201].

Single-cell RNA-sequencing has further revealed highly specialized monocyte subsets with distinctive functions in disease. Of these subsets, two newly identified *Cxcl10*^+^ and *Saa3*^+^ monocyte subsets expressing a pro-inflammatory signature were observed at the peak of disease [124]. These subsets were almost completely depleted by anti-CCR2 antibody administered at the peak of disease and correlated with an overall clinical improvement, suggesting these cells possess a characteristic pathogenic potential. Of note, these pathogenic monocyte subsets may not be derived from Ly6C^hi^ monocytes, since adoptive transfer of BM monocyte progenitors demonstrated that *Saa3*^+^ and *Cxcl10*^+^ monocyte subsets differentiated from granulocyte-monocyte progenitors and monocyte-macrophage DC progenitors evidently without passing through a Ly6C^hi^ monocyte state [124]. However, this transition is rapid during inflammation [73] and may have been missed at a 48 h time point, since Ly6C on monocytes and T cells is crucial for endothelial transmigration [44, 145, 146, 166, 310]. Nevertheless, this highlights the continuing development of our understanding of myeloid cell differentiation and responses in the pathogenesis of EAE and other neuroinflammatory diseases.

While monocytes evidently acquire a multitude of phenotypes in EAE, the inflammatory microenvironment appears to be critical in shaping the functional specialization of these subsets and their contribution to pathology. This may include cytokines, such as GM-CSF, that are produced by autoreactive T cells surrounding inflammatory lesions. Indeed, GM-CSF signalling is thought to induce a characteristic transcriptional signature in monocytes that is sufficient to drive disease onset [67, 190]. Monocytes with defective GM-CSF signalling do not participate in myelin destruction [135], suggesting the pathogenic activity of this subset is contingent on GM-CSF signalling by autoreactive T cells. Temporal and spatial factors in the inflammatory microenvironment further contribute to the functional specialization of infiltrating monocyte-derived cells. For instance, fate-mapping experiments of infiltrating phagocytes revealed that pro-inflammatory polarization (iNOS^+^) of these cells occurred predominantly in the spinal cord lesions [201] and perivascular space [177], whereas those expressing an anti-inflammatory phenotype (arginase-1^+^) resided predominantly in the meninges [164, 201]. iNOS^+^ cells expressing this characteristic pro-inflammatory signature predominated during the formation of lesions [201], but as lesions progressed towards resolution iNOS^+^ cells locally adapted their phenotype and transitioned towards an anti-inflammatory phenotype that was thought to guide EAE recovery [201]. When injected into the healthy CNS, these pro-inflammatory iNOS^+^ cells shifted their phenotype to an anti-inflammatory Arg1^+^ phenotype within a few days [201], strongly supporting the notion that the local CNS environment mediates this phenotypic switch. Indeed, in the absence of lesion resolution, monocyte-derived cells expressing an anti-inflammatory phenotype may return to a pro-inflammatory state in response to a chronic inflammatory environment [124], perhaps reflecting a transcriptional adaptation to the complex inflammatory lesion environment in which damage and regeneration are concurrent.

## Concluding remarks

CNS inflammation is an accompanying pathological feature of neurological diseases that can lead to neurodegeneration and progressive neurological deficits. Although multiple immune cell types can inflict injury within the CNS parenchyma, resident and infiltrating myeloid cells are key regulators of disease outcome. However, elucidating the precise contributions of each cell subset to neurological diseases has historically been hampered by the paucity of research tools available to accurately identify these cells in human and murine tissue. The development of new reagents and experimental systems has greatly improved our ability to distinguish resident from infiltrating myeloid cells in inflammation, unravelling the interplay and division of labor between these cell subsets in disease.

In summary, we have highlighted the non-redundant and often opposing mechanisms by which monocytes and microglia dampen or exacerbate immunopathology in murine and human studies of autoimmune neuroinflammation, CNS injury, and viral encephalitis. Although more human studies are required, findings from animal models suggest that both cell types can perform both protective and pathogenic functions, with microglia overall more protective in the acute phase of viral encephalitis and stroke, and seemingly more pathogenic in diseases with chronic neuroinflammation, including AD and MS, as well as in the recovery phase of viral infection. In contrast, the functions of monocyte-derived cells are not as clear-cut. These cells can perform protective functions that enhance viral clearance and tissue repair, however, and in general, monocyte infiltration occurs at the expense of detrimental tissue damage, albeit in many cases, priming the tissue for repair.

The inherent limitations of animal models must be considered when extrapolating findings to human disease. Nonetheless, this review has emphasized the critical importance of animal studies in understanding certain elements of CNS pathology. Currently translating our understanding of monocyte and microglial diversity into clinically useful therapeutic approaches remains a significant challenge. Accurately identifying temporally dependent myeloid phenotypes and their associated protective or pathological functions, both unique and common to various CNS pathologies, remains a crucial goal of current research. The Grail is in the precise, confident tailoring and harnessing of this specific knowledge.
